# The *transformer-2* and *fruitless* characterisation with developmental expression profiles of sex-determining genes in *Bactrocera dorsalis* and *B. correcta*

**DOI:** 10.1038/s41598-020-74856-6

**Published:** 2020-10-21

**Authors:** Kamoltip Laohakieat, Siriwan Isasawin, Sujinda Thanaphum

**Affiliations:** Regional R&D Training Center for Insect Biotechnology (RCIB), Department of Biotechnology, Faculty of Science, Mahidol University, Rama VI Road, Bangkok, 10400 Thailand

**Keywords:** Genetics, Gene expression

## Abstract

Sex determination in tephritid fruit flies involves a signaling cascade of alternatively spliced genes. The Transformer (TRA) and Transformer-2 (TRA-2) complex establishes an autoregulatory loop switching sex-specific splicing of *tra* pre-mRNA in females. The TRA/TRA-2 complex also regulates the sex-specific splicing of downstream effector genes, *doublesex* (*dsx*) and *fruitless* (*fru*). In *Ceratitis capitata*, a *Maleness-on the-Y* (*MoY*) gene modulates sex-specifically spliced *Cctra* pre-mRNA and results in the breakdown of the *Cctra* autoregulatory loop in males. In this study, the *tra-2* and *fru* genes were characterised in two key pests, *Bactrocera dorsalis* and *B. correcta.* The *tra-2* genes showed high degrees of conservation among tephritids. The complex gene organisation for each of *Bdfru* and *Bcfru* were identified. There are sex-specific and non sex-specific transcripts generated by alternative promoters as found in *Drosophila melanogaster* and other insects. RNAi knockdown of *Bdtra* transcripts showed that BdTRA controls the sex-specific splicing of *Bddsx* and *Bdfru* pre-mRNAs. Developmental expression analysis shows that multiple splice variants of *Bdtra* and *Bctra* RNAs are present before and during cellular blastoderm formation and that the mature sex-specific variants become fixed later in embryogenesis. Furthermore, the *Bddsx*^*M*^ splice variants are found in early embryos at the beginning of gastulation, but *Bdfru*^*M*^ does not appear until the larval stage. We proposed that the zygotic *tra* loop is initiated in both female and male embryos before becoming automatised or abolished by *MoY*, respectively.

## Introduction

Sexual reproduction is a key point for the continuation of multicellular eukaryotes. Males and females evolve into sexual dimorphism through the sexual selection processes^1^. Sex-determination mechanisms are highly diverse, and they usually occur during early development^2^. The sex determination of insects is one of the most diverse and well-characterised systems^3,4^. Its evolutionarily divergent model constitutes three levels of sex-determining regulators^4^. The primary signals are variable among insect species. Most of their signaling mechanisms are still unknown, but the signals are relayed via alternative splicing of a conserved binary switch gene. This switch gene, encoded for a splicing regulator, relays similar alternative splicing messages to the highly conserved downstream effectors.

Tephritid fruit flies are in a very diverse dipteran family comprising at least 5,000 species^5^. Several of them are high profile pests, including *Ceratitis capitata* (Mediterranean fruit fly), *Bactrocera dorsalis* (oriental fruit fly), *B. oleae* (olive fruit fly), *B. tryoni* (Queensland fruit fly), *Anastrapha ludens* (Mexican fruit fly), *A. obliqua* (West Indian fruit fly), and *A. suspensa* (Caribbean fruit fly). Many sex-determining orthologues have been characterised to understand the developmental cascades which are also beneficial for determining new pest control strategies^6–8^. For instance, genetic sexing strains have been developed by employing modern genetic approaches for pest control programs using the sterile insect technique (SIT)^6,9–13^.

Recently, the primary signal *Maleness-on the-Y* (*MoY*) has been discovered in *C. capitata*^14^. The *MoY* orthologues were also characterised in many *Bactrocera* spp. such as *B. dorsalis*, *B. oleae*, and *B. tryoni*. Disruption or overexpression of *CcMoY* can feminise XY embryos or masculinise XX embryos, respectively. *CcMoY* modulates sex-specific splicing of *transformer* (*tra*) pre-mRNA^14^. In tephritid female flies, the *tra* and *transformer-2* (*tra-2*) orthologues encode the TRA and TRA-2 proteins that later form a TRA/TRA-2 complex to maintain an autoregulatory loop of the female-specifically spliced *tra* transcript (*tra*^*F*^)^15–25^. The *Bdtra*^22–24^ and *Bdtra-2*^22,23^ were previously characterised in *B. dorsalis*. The RNAi-mediated knock-down of any one of these genes generated only males because the autoregulatory loop was disrupted. Likewise, the autoregulatory loop is disrupted by the presence of *MoY* orthologues in males^14^. The TRA/TRA-2 complex also switches on the Doublesex^F^ protein (DSX^F^) by regulating female-specific splicing of *dsx* pre-mRNA. The switching-off mode results in DSX^M^ because the absence of the functional TRA/TRA-2 complex allows male-specific splicing of *dsx* pre-mRNA^15–19,22–24^. DSX^F^ and DSX^M^ are highly conserved functional effectors in insects^26,27^. They are transcriptional factors with a masculinised or feminised function for downstream genes, controlling somatic sexual dimorphism^26,28^. The *dsx* orthologues studied in several tephritid fruit flies (i.e., *C. capitata*^29^, *B. tryoni*^30^, *B. oleae*^31^, *B. dorsalis*^32,33^, *B. correcta*^33^, and *A. obliqua*^34^) are conserved, suggesting similar somatic sexual differentiation among them.

In a tephritid and remotely related insects, the TRA/TRA-2 complex switches were also found to control the sex-specific splicing of the other downstream effector gene, *fruitless* (*fru*), that confers the sexual behaviors of males^18,35–38^. However, the *fru* gene has not been characterised in detail among tephritid species. The molecular mechanisms of *fru* in sex determination were proposed based on non-tephritid models^35–41^. The *fru* genes are very complex loci encoding sex-specific and non sex-specific transcriptional factor isoforms^42,43^. FRU proteins generally contain a dimerisation-interface domain called the BTB (Broad-complex, Tramtrack and Bric-a-brac) domain and one alternative C_2_H_2_ zinc finger domain^35,36,44,45^. Each of the *fru* orthologues has many putative transcripts derived from any one of the alternative promoter-derived 5′ sex- and non sex-specific exons, which connect to four to five common exons and then connect to any one of the 3′ alternative zinc-finger encoding exons^35,36,38–41^. Notably, the sex-specifically spliced transcripts are only processed from the most distal promoter (P1) derived pre-mRNA in many non-tephritid species^35,36,38,39,41^. In *Drosophila melanogaster*, the non sex-specific FRU isoforms have developmental functions. They are translated from P2 to P4 transcripts^46–48^.

In this study, we report the gene structure and expression of *tra-2* and *fru* orthologues in *B. dorsalis* and *B. correcta*. The RNAi knockdown experiments showed that BdTRA controls the sex-specific splicing of *Bdtra*^*M*^, *Bddsx*^*M*^, and *Bdfru*^*M*^ transcripts in pseudomales. In addition, a comprehensive sex-determination cascade was investigated from the developmental expression profiles of *MoY*, *tra*, *tra-2*, *dsx*, and *fru* in single embryos, larvae, pupae, and adults. These results likely ascertained the time frames of critical sex-determining events in these *Bactrocera* fruit flies.

## Results

### *B. dorsalis* and *B. correcta transformer-2* (*tra-2*) orthologues

**Figure 1:**
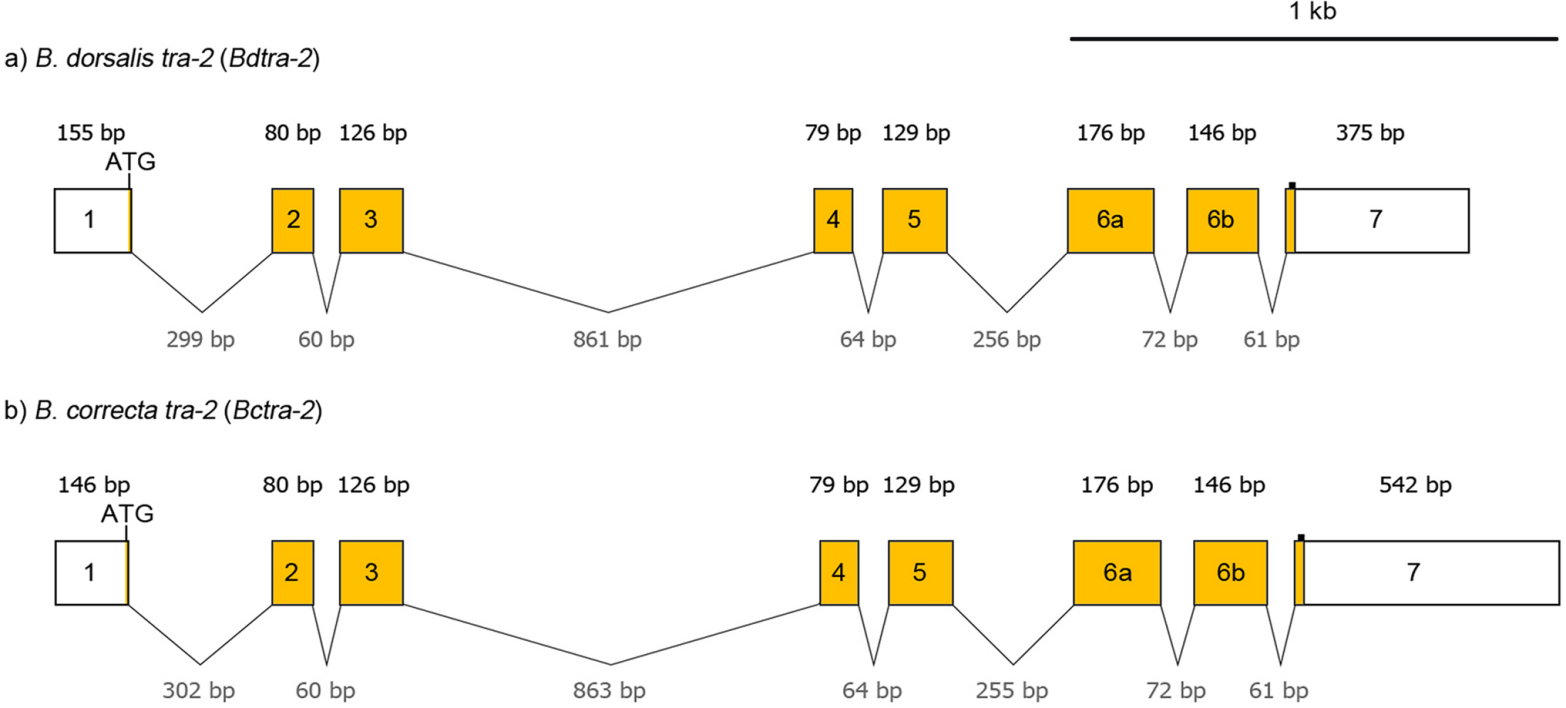
Molecular structure of *tra-2* genes from (**a**) *B. dorsalis* and (**b**) *B. correcta*. Both genes contain eight exons. Boxes and lines represent exons and introns, respectively. The white and yellow parts in the exons are untranslated and translated regions, respectively. The potential start codons (ATG) are indicated. Small black squares mark the potential stop codons.

Full-length transcripts of *B. dorsalis tra-2* (*Bdtra-2*) and *B. correcta tra-2* (*Bctra-2*) are approximately 1.2 kilobases (kb) and 1.4 kb, respectively, in both sexes. The genomic DNA and cDNA sequences of *Bdtra-2* (GenBank Acc. No. MT900512, MT900514) and *Bctra-2* (GenBank Acc. No. MT900513, MT900515) were acquired from adult males and females. Both genes consist of eight exons (Fig. 1) and are structurally comparable to *B. oleae* (GenBank Acc. No. AJ547623), *B. tryoni*^21^, *B. jarvisi*^21^, *Z. cucurbiate*^25^*, C. capitata*^18^, *Anastrepha* spp.^19,20^, and *D. melanogaster*^49,50^. The open reading frames (ORF) of *Bdtra-2* and *Bctra-2* are 753 bp, encoding for a predicted polypeptide of 251 amino acids with 98% identity (Supplementary Table S1). The RNA recognition motif (RRM) region of BdTRA-2 and BcTRA-2 were identical within *Bactrocera* species and highly conserved across the tephritidae family (Supplementary Fig. S1). The nucleotide sequences of the *Bdtra-2* are almost identical to the previously characterised *Bdtra-2*^22^; however, 29 bp-longer and 88 bp-shorter sequences were found at the 5′- and 3′-UTRs, respectively.

### *B. dorsalis* and *B. correcta fruitless* (*fru*) orthologues

**Figure 2:**
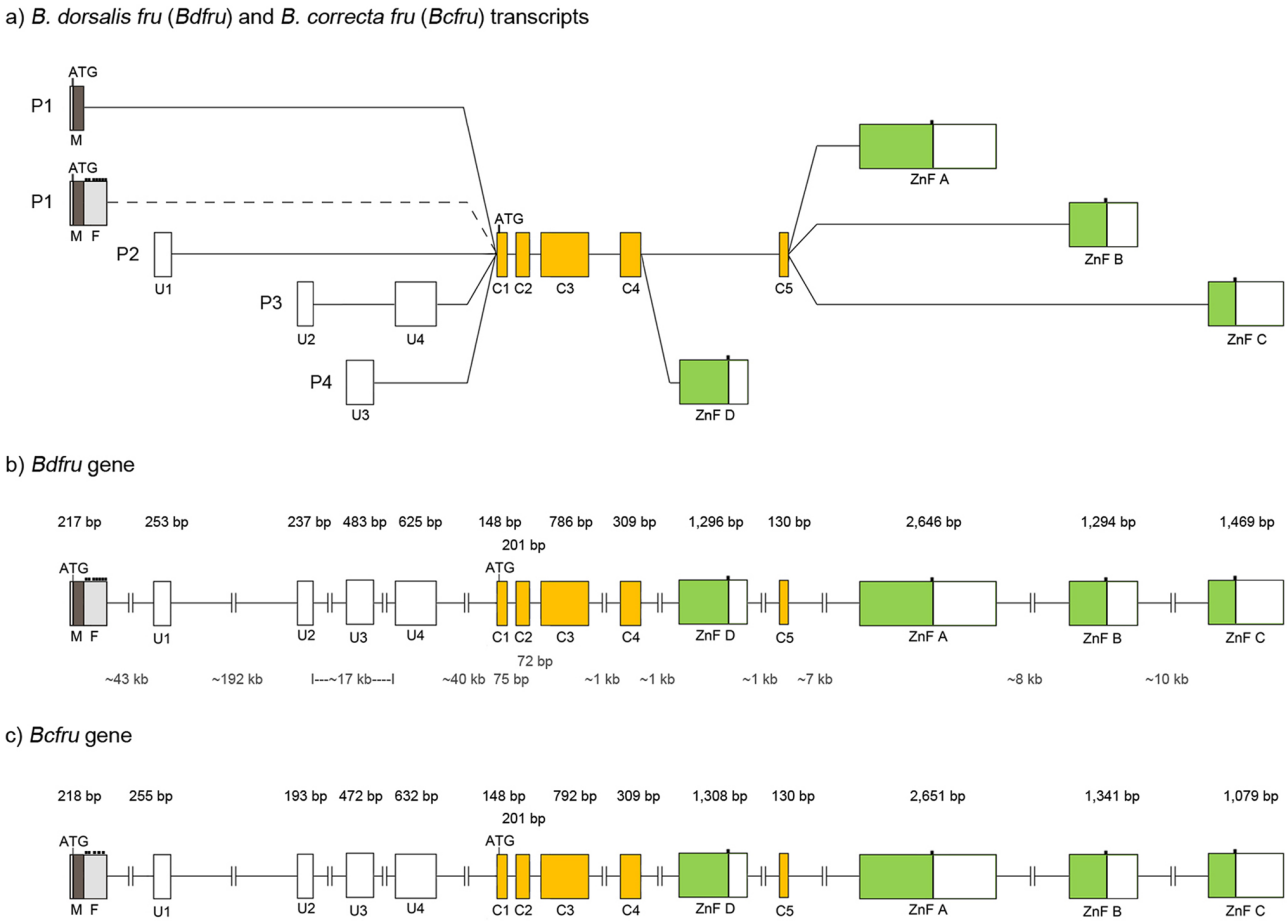
Gene organisation of *B. dorsalis fru* (*Bdfru*) and *B. correcta fru* (*Bcfru*) genes. The schematic drawings show (**a**) all possibilities for alternative splicing of *fru* transcripts and gene organisation of (**b**) *Bdfru* and (**c**) *Bcfru* (not to scale). The *fru* transcripts contain one of the four alternative 5′ exon blocks (i.e., sex-specific, U1, U2–U4, and U3 blocks), the common exons (i.e., C1 to C5 exons), and four alternative 3′ ends (i.e., ZnF A to ZnF D exons). P1 signifies the most distal promoter; its transcripts begin with a male-specific exon (M) and potential female-specific exon (F) as shown in dark gray and light gray boxes, respectively. P2 to P4 express non sex-specific transcripts which start with untranslated exons (U1–U4) as shown in the white boxes. The five common exons (yellow boxes) are the BTB domain coding exons (C1–C2) and the connecting exons (C3–C5). The zinc-finger encoding exons (ZnF A, ZnF B, ZnF C, and ZnF D) are in the green boxes. Boxes and lines represent exons and introns, respectively. Two putative translational start codons (ATG) are presented in the M and C1 exons. The potential translational in-frame stop codons are indicated by small black squares. The white and coloured parts in the exons represent untranslated and translated regions, respectively. The lengths of exons and putative introns are shown in black and gray numbers, respectively. The connection between F and common exons (dashed line) is still hypothetical because the RT-PCR experimental results were not positive.

A classical PCR-based approach was combined with available bioinformatics tools and genomic databases to predict the putative exon–intron organisation of the *fru* gene. The 5′-, 3′-RACEs and RT-PCRs were performed to assemble long transcripts of *B. dorsalis fru* (*Bdfru*) (GenBank Acc. No. MT900516–MT900519, MT900524–MT900528) and *B. correcta fru* (*Bcfru*) (GenBank Acc. No. MT900520–MT900523, MT900529–MT900533) genes. Both orthologues consist of three components (i.e., alternative P1 to P4 derived exons, the middle common exons, and four alternative 3′ ending exons (Fig. 2a and Supplementary Fig. S2). For *B. dorsalis*, the genomic sequence is available in the database; cDNA sequences of *Bdfru* transcripts were aligned against the genomic scaffold (GenBank Acc. No. NW_011876307) to analyse the gene organisation. The results suggested that the *Bdfru* gene structure is very large in size, spanning approximately 330 kb of genomic sequence (Fig. 2b). The genome sequence of *B. correcta* is not available. The putative *Bcfru* structure was thus predicted based on cDNA sequence and conceptual amino acid sequence alignments of *Bcfru* and *Bdfru* transcripts, putative donor–acceptor exon sequences, and conceptual amino acid position. The results showed the identical pattern of putative donor–acceptor nucleotide sequences on exons and conceptual amino acid sequences. This suggested that the predicted exon structure of *Bcfru* gene is similar to that of *Bdfru* gene (Fig. 2c and Supplementary Fig. S2). However, the availability of *B. correcta* genomic sequences would bring certainty to the proposed *Bcfru* gene organisation.

At the 5′ portions of the *Bdfru* and *Bcfru* genes, five putative exons (male-specific exon (M) and untranslated exons (U1, U2, U3, and U4)) were found and spliced to the middle common exons in four different patterns (Fig. 2a and Supplementary Fig. S2). The genomic location of the four alternative 5′ exons are located many kb apart. This suggested that there are at least four putative promoters. The most distal promoter of *Bdfru* transcripts begin with an M exon, which is located 292 kb upstream of the C1 exon and appears to be a male-specifically spliced transcript (P1 transcripts) (Fig. 2b). The other three patterns are non sex-specific transcripts as P2 transcripts begin with the U1 exon located 249 kb upstream of the C1 exon, P3 transcripts begin with the U2 exon located 57 kb upstream of the C1 exon, and P4 transcripts begin with the U3 exon located 49 kb upstream of the C1 exon. The common C1–C2 and C3–C5 exons encode for the BTB domain and its connecting polypeptides, respectively (Fig. 2a). Additional complexity arises from the presence of four alternative 3′ end exons, which are zinc-finger encoding exons (ZnF A, ZnF B, ZnF C, and ZnF D) (Fig. 2a). The 3′ RACE and RT-PCR approaches were used to analyse the connection between each of the P1 to P4 derived exons and the alternative zinc-finger exons of *Bdfru* and *Bcfru* (Supplementary Table S2). The alternative ZnF A, ZnF B, and ZnF C exons were part of the P1 transcripts whereas the P2 and P3 transcripts consisted of all possible alternative zinc-finger exons. The P4 transcripts included the alternative ZnF A, ZnF C, and ZnF D exons. In addition, the M exon provides an extra start codon in the ORF resulting in an additional 64 amino acids at the amino terminal end of male-specifically spliced P1 transcripts (Supplementary Fig. S3). The other four exons (U1 to U4) are untranslated regions; therefore, ORFs of the P2, P3, and P4 transcripts may be translated with the putative start codon located in the BTB coding exon (C1).

### In silico analysis of the female-specific exon and its *cis*-regulatory elements in *Bdfru* and *Bcfru* transcripts

To identify the sex-specific transcripts that were potentially derived from the P1 promoter, the forward primer located on the M exon and the reverse primer located on the common BTB exon C2 were used. A longer amplified product was expected to be detected in female flies due to the presence of a female-specific exon as reported in the other dipteran insects^35–41^. By contrast, in *B. dorsalis* and *B. correcta*, the amplified RT-PCR product was only observed in males. The bioinformatics approach was then used to identify the putative female-specific (F) exon and its *cis*-regulatory elements from the genomic scaffold. The 1.9 kb region downstream of the M exon was available in the *B. dorsalis* genomic scaffold (GenBank Acc. No. NW_011876307.1). The putative TRA/TRA-2 binding sites and multiple in-frame stop codons were in silico identified in this region (Supplementary Fig. S4). Both components were found to be clustered in an approximately 400 bp stretch adjacent to the M exon. PCR was performed using genomic DNA templates to verify this 400-bp region in *B. dorsalis* and *B. correcta*. The putative TRA/TRA-2 binding sites and multiple in-frame stop codons were found in *B. dorsalis* as expected and also in *B. correcta* (Supplementary Fig. S4a and S4b). The putative TRA/TRA-2 binding sites of *Bdfru* and *Bcfru* genes were comparable to the consensus sequence of TRA/TRA-2 binding sites located in the female-specific exon of the *fru* genes from the other insect species (Supplementary Fig. S4c). The in silico analysis suggested that the 400-bp region has the structural features of the putative F exon of *Bdfru* and *Bcfru* transcripts.

RT-PCR experiments were subsequently performed to prove the existence of the F exon (Supplementary Fig. S5). The cDNA templates were prepared from male and female adult heads of both species. Three forward primers (located on the M exon, putative junction of the M and F exons, and the putative F exon) were individually paired with a reverse primer (located on the putative F exon) (Supplementary Fig. S5a). The RT-PCR experiment showed a female-specific band when these primer combinations had been used (Supplementary Fig. S5b). This result suggested the existence of an F exon which is adjacent to 3′ end of the M exon. The connection between the F exon, and the common exon C2 could not be validated by the use of a C2-specific reverse primer in RT-PCR experiments (Supplementary Fig. S5d). Taken together, the in silico analysis and RT-PCR experiments suggested that the female-specific *Bdfru* and *Bcfru* transcripts contain potential in-frame stop codons that result in prematurely truncated female-specific ORF.

### *Bdtra* gene regulated sex-specifically spliced *Bddsx* and *Bdfru* transcripts

**Figure 3:**
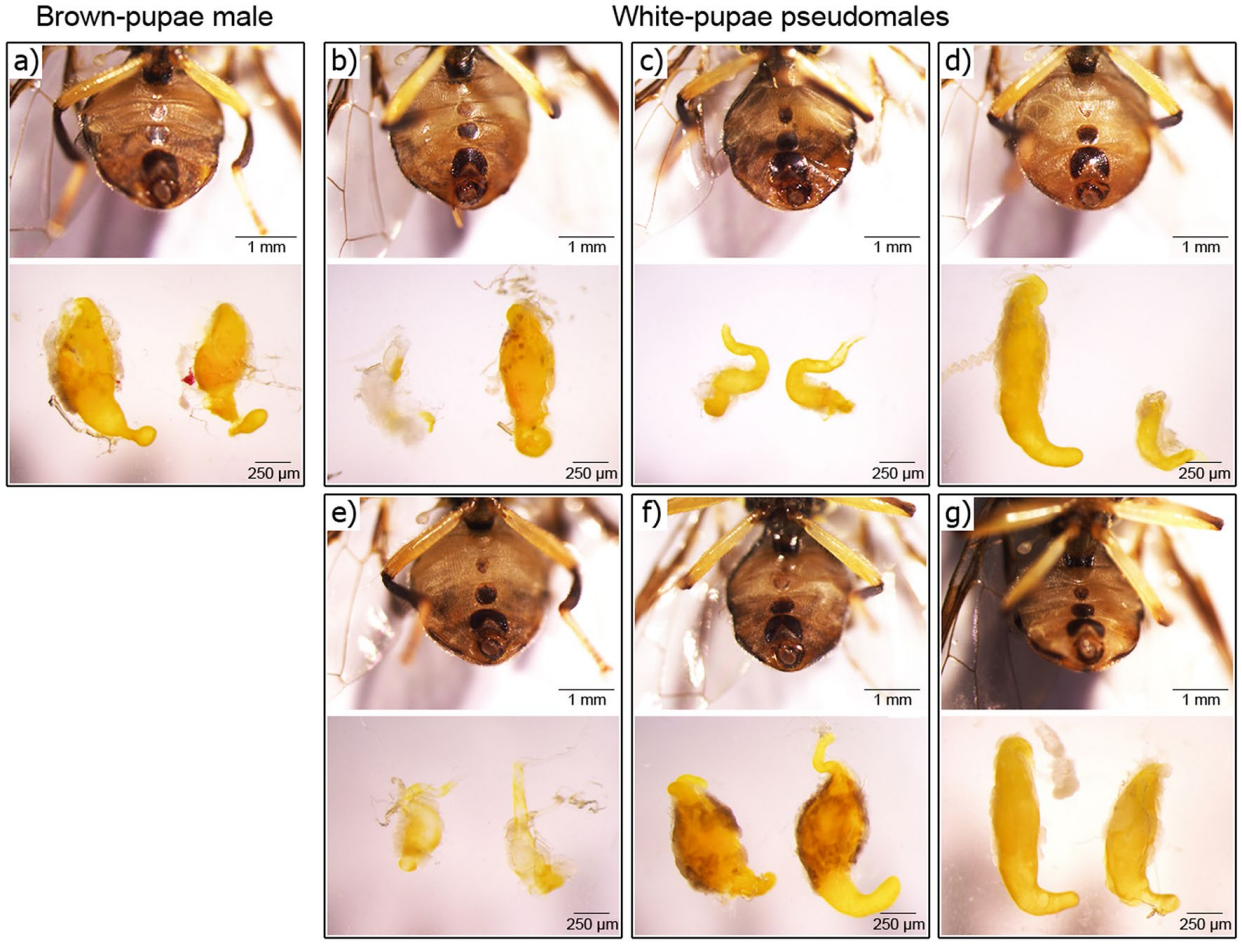
Phenotypic analysis of male genitalia and testes development of *Bdtra* dsRNA-treated pseudomales. The morphologically male external genitalia and dissected testes are photographed from the *Bdtra* dsRNA-treated (**a**) brown-pupae male and (**b**) to (**g**) white-pupae pseudomales.

**Figure 4:**
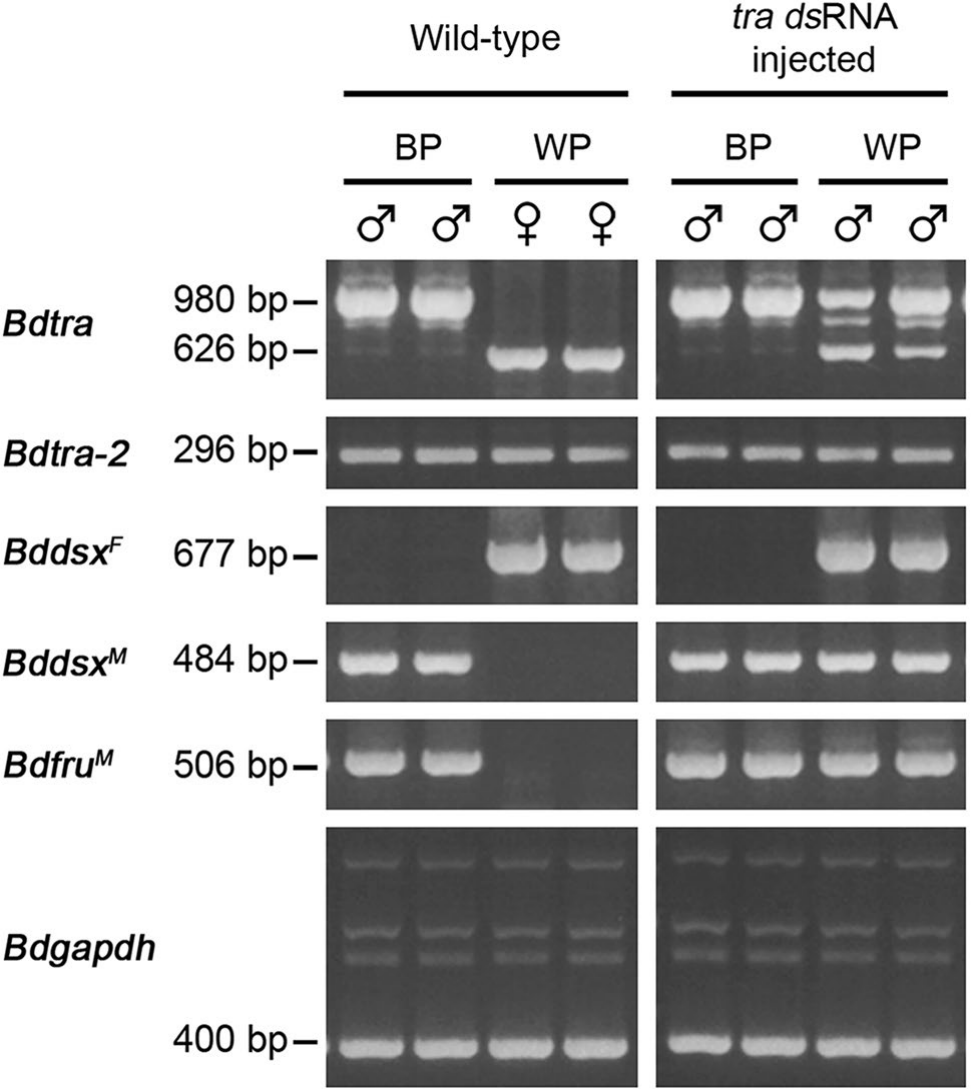
Expression analysis of *Bdtra* dsRNA treated and wild-type *B. dorsalis*. RT-PCRs of *Bdtra*, *Bdtra-2*, *Bddsx*, and *Bdfru* were performed to analyse the switch of splicing patterns from female to male resulting from the masculinisation by *Bdtra* dsRNA injection. The expression of *Bdgapdh* is used as a positive control. The expression patterns of the wild-type brown-pupae (BP) males and white-pupae (WP) females were compared with the *Bdtra* dsRNA treated brown-pupae males and white-pupae pseudomales. The primers used in this experiment are shown in Supplementary Tables S5 and S6. The full-length gels are presented in Supplementary Fig. S9.

The endogenous *Bdtra* transcripts were knocked down in preblastoderm embryos by the microinjection of *Bdtra* dsRNA. The brown- and white-pupae genetic sexing strain (Salaya1) of *B. dorsalis* was used to identify adult pseudomales (males emerging from white pupae) (Fig. 3). Male-specifically spliced *Bdtra, Bddsx*, and *Bdfru* transcripts were detected in the same adult pseudomales although the female-specific isoforms of the *Bdtra* and *Bddsx* were still observed in contrast to the sex-specific patterns observed in the male- and female-wild-types as well as the *Bdtra* dsRNA treated brown-pupae males (Fig. 4). *Bdtra-2* transcripts were detected in all cases. All of the adult pseudomales had male genitalia and mostly abnormal testes (Fig. 3). The results suggested that *Bdtra* RNAi can change the expression of *Bddsx* and *Bdfru* and generate masculinisation of the female embryos.

### The expression profiles of sex-determining genes during early embryonic, larval, pupal, and adult stages in the males and females of *B. dorsalis* and *B. correcta*

The early development stages of *B. dorsalis* and *B. correcta* embryos were very similar when observed from 0 to 9 h after egg laying (h AEL) (Supplementary Fig. S6). The pole cells and syncytial blastoderm appeared at 3 and 4 h AEL, respectively. Subsequently, the cellular blastoderm seemed to be completed by 7 to 8 h AEL.

**Figure 5:**
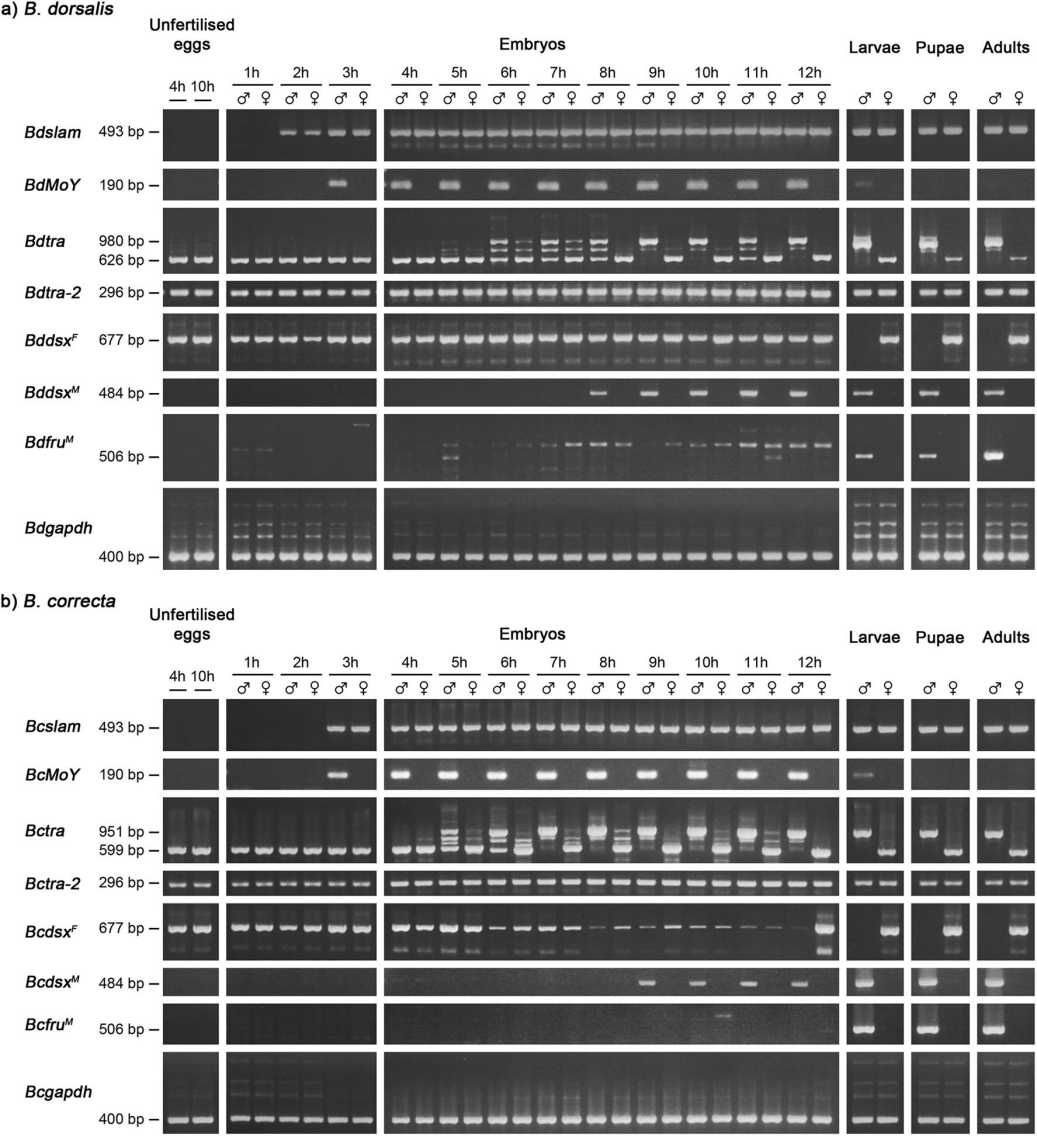
Expression analysis of sex-determining genes during the early stages of embryogenesis and other developmental stages in (**a**) *B. dorsalis* and (**b**) *B. correcta*. The cDNA samples were prepared from unfertilised eggs, embryos (1 to 12 h AEL), larvae, pupae, and adults. The presence of the *slam* gene indicates the zygotic transcription period in embryogenesis. Five sex-determining genes, *MoY*, *tra*, *tra-2*, *dsx*, and *fru*, were studied by RT-PCR analysis. In *B. dorsalis* (**a**), the male- and the female-specifically spliced transcripts (*tra*^*M*^ and *tra*^*F*^) are 980 bp and 626 bp, respectively. In *B. correcta* (**b**), the male- and the female-specifically spliced transcripts (*tra*^*M*^ and *tra*^*F*^) are 951 bp and 599 bp, respectively. For the *dsx* gene, the female-specifically spliced *dsx* transcript (*dsx*^*F*^) is 677 bp whereas the male-specific *dsx* transcript (*dsx*^*M*^) is 484 bp in both species. The male-specifically expressed *fru* transcript (*fru*^*M*^) appears in male samples at a length of 506 bp. Unexpected bands are the result of non-specific binding of primers. The *gapdh* gene was used as a positive control. The negative control was setup in the absence of RTase. The primers used in this experiment are shown in Supplementary Table S5 and S6. The full-length gels are presented in Supplementary Fig. S10.

The expression profiles of *MoY*, *tra*, *tra-2*, *dsx*, *fru*, and *slow as molasses* (*slam*) were studied in unfertilised eggs and during early embryonic development (1 to 12 h AEL) as well as in larval, pupal, and adult stages (Fig. 5). Gene- and/or sex-specific transcript primers were used for RT-PCR from single embryos, larvae, pupae, and adults. The embryos, larvae, and pupae were sexed by PCR using genomic DNA templates with Y-specific primers (Supplementary Table S6).

In *B. dorsalis*, the expression of *Bdslam* began in 2 h AEL zygotes and continued throughout the adult stage (Fig. 5a). The *slam* seemed to be the first zygotically expressed gene. The presence of female-specifically spliced *Bdtra* (*Bdtra*^*F*^) and *Bddsx* (*Bddsx*^*F*^), and *Bdtra-2* transcripts in unfertilised eggs shows that these transcripts are contributed maternally. The continued presence of the transcripts in developing embryos indicates zygotic activation of transcription of these genes. The expression of the *MoY* transcript was only observed in male samples beginning from 3 h AEL embryos, barely visible in larvae, and disappeared in pupae and adults. The combination of *Bdtra*^*F*^, zygotic male-specifically spliced *Bdtra* transcripts (*Bdtra*^*M*^), and their intermediate transcripts were firstly observed in both male and female samples at 5 h AEL. The intermediate transcripts consisted of three possible partial sequences of male-specific exon(s) (Supplementary Fig. S7). This observation became more obvious at 6 to 7 h AEL. This suggested that the activation of the zygotic *tra* loop had occurred in both sexes. In 8 h AEL embryos, the same combinations of the *Bdtra*^*F* and *M*^ and intermediate transcripts was observed in males. Conversely, the *Bdtra*^*F*^ transcripts were only a major product in female embryos, suggesting higher efficacy of the zygotic *tra* loop. Sex-specific splicing patterns of the *Bdtra* gene arose in 9 and 10 h AEL embryos. The female-specifically spliced pattern continued to at least 12 h AEL and in larvae, pupae, and adults. The male-specifically spliced counterpart also followed this trend, except that the mixture of *Bdtra*^*F* and *M*^ and intermediate transcripts reappeared once in the 11 h AEL male zygotes. The expression of the zygotic male-specifically spliced *Bddsx* transcript (*Bddsx*^*M*^) appeared at the beginning of gastrulation (8 to 10 h AEL) and was detected at all later stages in males. The sex-specific splicing pattern of the *Bddsx* gene was established in the larvae, pupae, and adults. The male-specifically spliced *Bdfru* transcript (*Bdfru*^*M*^) derived from the P1 promoter was observed in male samples from larval, pupal, and adult stages, but not in the embryonic stage. Notably, a non-specific RT-PCR amplicon was observed and sequenced. It was found to be against predicted *B. dorsalis* protein peanut and E3 ubiquitin-protein ligase genes in embryos when BLAST was used. The expression profiles of the other *Bdfru* transcripts derived from the P2, P3, and P4 promoters were also absent in the unfertilised eggs and embryos when tested in 4, 8, and 12 h AEL embryos (Supplementary Fig. S8a). Unlike the male-specifically spliced P1 transcripts, the other *Bdfru* transcripts were bisexually expressed in larvae, pupae, and adults. Nonetheless, all *Bdfru* transcript isoforms appeared during development later than the other sex-determining genes. All sex-determining orthologous genes from *B. correcta* showed similar expression profiles to those of the *B. dorsalis* species (Fig. 5b, Supplementary Fig. S7, and S8a). However, the appearance of *Bcslam* and *Bcdsx*^*M*^ occurred an hour after those of the *B. dorsalis*.

## Discussion

The molecular organisations of *Bdtra-2* and *Bctra-2* are almost identical to both the previously characterised *Bdtra-2*^22,23^ and orthologues from other tephritid fruit flies^18–20,25^, including *Bactrocera* spp.^21^. TRA-2 is a constitutive component required for TRA/TRA-2 complex for turning on the sex-determining-autoregulatory loop that generates female-specifically spliced *tra* transcripts in tephritids^18–20,22^. The TRA-2 proteins have two conserved RS-rich regions and an RRM domain which were proposed to be under the purifying selection process^19,25^ (Supplementary Table S1 and Fig. S1). This suggested the roles of TRA-2 in spliceosome formation^49,51,52^.

This work is the first study of *fru* genome organisation with various splicing patterns and expression profiles during development in tephritid fruit flies. The *Bdfru* and *Bcfru* orthologues are also one of the longest and most complex genes. The *Bdfru* gene spans approximately 330 kb. There are probably many transcripts with conserved splicing patterns, as in many insect species: *D. melanogaster*^35,36^, *Anopheles gambiae*^39^, *Aedes aegypti*^41^, *Musca domestica*^38^, and *Nasonia vitripennis*^40^. Alternative promoters can generate various 5′ exons that connect to the continuous array of four or five common exons before splicing with one of the different 3′ zinc-finger encoding exons.

The overall deduced amino acid sequences of the BdFRU and BcFRU are generally conserved, as in *D. melanogaster*. The BTB domain is the most conserved characteristic of all FRU proteins^53,54^. It is encoded near the beginning of the C1 exon. This BTB domain is an evolutionarily conserved protein–protein interaction domain found throughout the evolutionary lineages from *Drosophila* to mammal^44^. Many alternative zinc-finger domains for different FRU proteins are less conserved. On the other hand, a high degree of polymorphism was found in amino acid sequences encoded by the male-specific exon. This male-specific sequence is still highly conserved within the family (i.e., tephritidae). This suggests the strong adaptive divergence of sexual behaviors and may confer male-specific transcriptional regulatory roles of FRU^M^ in different insect taxa.

The P1 derived primary transcripts of *Bdfru* and *Bcfru* are sex-specifically spliced at the 5′ exons as in the case of *D. melanogaster*^35,36^, *An. gambiae*^39^, *Ae. Aegypti*^41^, *M. domestica*^38^, and *N. vitripennis*^40^*.* The male- and female-specific exons are adjacent. TRA/TRA-2 binding sites located in the female-specific exon suggested an alternative splicing mechanism which selected the 5′ splice site located at the end of the female-specific exon. The binding of TRA/TRA-2 complex hinders the formation of the spliceosome complex at the male-specific splice site in *D. melanogaster*^37^. The female-specific FRU protein was truncated because of its in-frame stop codons in the female-specific exon^36–41,45,46^. Transcripts connecting the female-specific exon and the common C1 exon could not be confirmed by RT-PCR experiments because the female-specifically spliced transcript may be expressed at a low level. It was found that the expression level of the *fru*^*F*^ transcripts were lower than the *fru*^*M*^ transcripts in *D. melanogaster*^46^ and *Ae. Aegypti*^41^.

We propose that *Bdfru* and *Bddsx* are regulated by *Bdtra* in the sex-determination pathway as previously proposed in the Medfly^15,18^. This is based on the presence of TRA/TRA-2 binding sites in their female-specific exons^22–24,32,33^ (in this study) and the sex-determining gene splicing analysis in the pseudomales made from *Bdtra* RNAi. The same regulatory roles of *Bctra* to the *Bcfru* and *Bcdsx* genes can be implicated based on the conserved sequences and gene structures^24,33^ (Fig. 2) and developmental expression profiles (Fig. 5).

The expression profiles of sex-determining genes in *B. dorsalis* and *B. correcta* begin with maternal *tra*^*F*^ and *tra-2* transcripts. The maternal-to-zygotic transition of these genes may have occurred at 2–3 h AEL as indicated by the expression of the zygotic cellularisation *slam* gene. In *B. jarvisi* and *C. capitata*, the expression of the orthologs of the *slam* gene were detected in embryos as early as 4 h AEL^21,55^. The Y-linked *BdMoY* and *BcMoY* genes also started to express around the 3 h AEL in male embryos. In *C. capitata*, the *CcMoY* transcription was also detected between 2–3 h AEL^14^. All of these *MoY* orthologues continued to express in males during the embryonic stages, less so in the larvae, and disappeared in the pupae and adults^14^ (in this study).

The expressions of zygotic *Bdtra* and *Bctra* genes were firstly indicated by the appearance of the *Bdtra*^*M*^ and *Bctra*^*M*^ transcripts at 5 h AEL for both male and female embryos. However, these transcripts were found in heterogeneity, i.e., all together with *Bdtra*^*F*^ and *Bctra*^*F*^ and their intermediate transcripts. The heterogeneous pattern of RNA splicing was equally extended from 5 to 8 h AEL in both sexes. This finding is consistent with the expression pattern of *Cctra* during the same developmental period^55^. It is hypothesised that the activation of the zygotic *tra* loop occurred in all embryos during this period. For the XX embryos, the accumulation of TRA/TRA-2 complex level above a certain threshold would generate a *tra*-autoregulatory loop. TRA and TRA-2 are serine/arginine (SR) type splicing regulators which operate under threshold dependency^56^. The presence of *Bdtra*^*F*^ and *Bctra*^*F*^ and the absence of *Bdtra*^*M*^, *Bctra*^*M*^, and their intermediate transcripts suggested that the level of TRA/TRA-2 complex was high enough to complete the automatisation of the *tra* loop from the 9 h AEL developmental point onward in females. In the case of the XY embryos, the non-autonomous zygotic *tra* loop was later disrupted by the suppression of the *MoY* gene. The appearance of mostly *Bdtra*^*M*^ and *Bctra*^*M*^ transcripts suggested the breakdown of the *tra* loop in males beginning at 9 h AEL. This finding is supported by a new discovery of an autosomal derived microRNA, miRNA-1-3p, that is required for male determination of *B. dorsalis*^57^. The miRNA-1-3p is involved in the suppression of *Bdtra* transcripts by targeting 3′ UTR. The relative expression of this miRNA-1-3p is also significantly increased and male-biased during the same critical period of the *Bdtra* loop breakdown process, i.e., from 6 to 9 h post oviposition at 28 °C. The microRNA is an example of intermediate male determiners. Further bioinformatics and molecular studies that shed light on how *MoY* relays the instruction to disrupt the *tra* loop will be focused on the period of 5 to 9 h AEL in *B. dorsalis* and *B. correcta* embryos.

The *Bddsx* and *Bcdsx* genes are maternally transcribed. The *Bddsx*^*F*^ knockdown experiment resulted in an interruption of *yolk protein* (*yp*) expression in adults and led to a significant reduction of ovary size, number of oocytes, and malformation of reproductive organs^32^. The presence of *Bddsx*^*F*^ and *Bcdsx*^*F*^ transcripts were observed in both sex embryos up to at least 12 h AEL. DSX^F^ may be used in the expression of genes related to the nourishment of embryos such as the *yp* gene^58^. A similar finding showed that the *Bjdsx*^F^ transcript was also expressed in both sex embryos at sometime between 1 to 9 h AEL^21^. In the medfly, the *Ccdsx*^*F*^ transcription was maternally derived and also detected at 4 h AEL in all embryos. However, the *Ccdsx*^*F*^ disappeared from 5 to 9 h AEL and reappeared as a pattern of sex-specifically spliced *Ccdsx*^*M*^ and *Ccdsx*^*F*^ transcripts in male and female embryos, respectively, at 10 h AEL^55^. This indicated that the zygotic expression of the *Ccdsx* gene had begun around this time. The first zygotic expression of *Bddsx* and *Bcdsx* genes were also in evidence at 8 and 9 h AEL, respectively, when the *Bddsx*^*M*^ and *Bcdsx*^*M*^ transcripts were detected. In *B. jarvisi*, a high number of *dsx*^*M*^ transcripts was detected after 6 h AEL^21^.

If the presence of *Bddsx*^*F*^ and *Bcdsx*^*F*^ transcripts were zygotically expressed in male embryos during the period of 9 to 12 h AEL, it would result from the binding between TRA/TRA-2 complex proteins and the *dsx* pre-mRNA. However, there is evidence that the availability of functional TRA/TRA-2 complex protein is not enough to generate *Bdtra*^*F*^ and *Bctra*^*F*^ transcripts from their pre-mRNAs for the same duration. This suggests that TRA/TRA-2 binding complexity at the respective pre-mRNAs of the *tra* and *dsx* orthologues are different. Ruiz et al.^17^ proposed, due to the putative *cis*-regulatory elements on sex-specific exons of *tra* and *dsx* pre-mRNAs, that TRA plays a dual role in the sex-determination pathway of tephritids. The putative TRA/TRA-2 complex binding sites on the female-specific exon of *dsx* pre-mRNA suggested the requirement of simpler components for the formation of an enhancing complex. In contrast, additional TRA-2 and SR protein(s) may be required for binding at the putative silencing elements on the male-specific exon(s) of *tra* pre-mRNA^17^.

The first appearance of *Bddsx*^*M*^ and *Bcdsx*^*M*^ transcripts in cellular blastoderm embryos (from 7 to 9 h AEL, supplementary Fig. S6) suggested the presence of both effector proteins, DSX^M^ and DSX^F^. This indicated that sex-specific transcription of DSX-regulated genes could begin in the embryo. However, the sex-specifically spliced patterns were distinctively observed in larval, pupal, and adult stages where sexual dimorphism are well developed and require regulation of later sexual differentiation genes.

The *fru* gene is the other downstream effector required for later sexual differentiation. The developmental expression of the P1 derived *Bdfru* and *Bcfru* transcripts were detected later in the larval, pupal, and adult stages similar to that of *D. melanogaster*^36^ and *Ae. aegypti*^41^. In *D. melanogaster* males, default splicing of P1 transcripts are expressed in a small fraction of neurons^36,46,59–61^. This results in functional FRU^M^ proteins playing transcriptional factor roles which determine male courtship and orientation^59,60,62^. This suggests that the FRU^M^ effector influences male development of neurons and sexual behaviors after the embryonic stage. The P2 to P4 derived *Bdfru* and *Bcfru* transcripts were also detected in a similar developmental stages to the P1 transcript. They may have a non sex-specific developmental function as found in *D. melanogaster*^36,46–48^. Further functional studies of the *fru* genes could be complex because there are a lot of *fru* transcript variants. However, the function of the *fru*^*M*^ transcripts would be significantly related to the sex-determination pathways, sexual development of the insect brain, and sexual behavior.

The molecular study of *Bdtra-2* and *Bdfru* genes have made it possible to investigate every player in the general sex-determination cascades of tephritid fruit flies^4^: *BdMoY*^14^, *Bdtra*^22–24^, *Bdtra-2*^22,23^ (also in this work), *Bddsx*^32,33^ and *Bdfru* in *B. dorsalis*. The developmental expression profile analyses can outline the following critical time points. The presence of maternal *Bdtra* transcripts could provide BdTRA proteins to initiate the autoregulatory loop. The *Bdtra-2* transcript is continuously expressed throughout development as found in a previous study^22^. The Y-linked male-determiner, *BdMoY*, starts expressing instructions from 3 h AEL until the larval stage. The zygotic *tra* loop activation becomes apparant in both sex embryos at 5 h AEL. The first male-specific splicing pattern of *Bdtra* suggests that the BdMOY protein has completed the process of *Bdtra* loop disruption at 9 h AEL. In addition, the *Bdtra* autoregulatory loop is fully functional due to the disappearance of *Bdtra*^*M*^ and intermediate transcripts from 8 to 9 h AEL in female embryos. When the binary *Bdtra* is switched OFF, it transduces the first signal to the *Bddsx*^*M*^ effector at 8 h AEL although the appearance of the *Bddsx*^*F*^ transcript is present in male embryos until at least 12 h AEL. The expression of the BdFRU^M^ effector is not apparent in embryos but only in later development. The comparative studies of all sex-determining orthologues from *B. correcta* imply similar sex-determination schemes^24,33^ (in this study). Detailed study of sex-determination cascades can be focused on more specific times in embryonic and sexual development of tephritid fruit flies. Fundamental mechanisms (e.g., how *MoY* breaks down the *tra* loop; how the autoregulatory loop of zygotic *tra* is established; how *tra* switches ON and OFF sex-specific splicing of *dsx* and *fru* pre-mRNAs; and how *fru* + neurons program male sexual behaviors) can be explained with better resolution. Novel intermediates and accessory proteins required for sex-determination can be screened and validated using bioinformatics and genome editing approaches. Comprehensive understanding of sex-determination may lead to new genetic tools and the improvement of insect pest control strategies in the high profile key pests, *B. dorsalis* and *B. correcta*^63^.

## Methods

### Fruit fly strains

The laboratory strains, *B. dorsalis* (Phayathai1 strain) and *B. correcta* (Phayathai2 strain), were used for the isolation of *fru* and *tra-2* genes and the expression analyses of sex-determining genes in different developmental stages. The *B. dorsalis* Salaya1 genetic sexing strain was used for the RNAi experiment of *Bdtra*. This strain has sexual phenotypes of brown/white pupae, with males and females emerging from brown and white pupae, respectively^64^. The fruit flies were reared at 25 °C, with 13 L: 11 D cycles, and around 70% relative humidity (RH) at Regional R&D Training Center for Insect Biotechnology, Mahidol University.

### Egg and embryo collection

Two different fruit fly cages were set up for harvesting unfertilised eggs and embryos from sexually mature (14-day old) virgin and mated females, respectively. The induction of egg oviposition was done by putting a pretested oviposition chamber containing guava juice into the fruit fly cages for one hour. The experimental batches of eggs and embryos were harvested from each new oviposition chamber after being left in the cage for 10 min. All samples were incubated at 25 °C and 70% RH. The first batch of eggs was incubated for 48 h to validate virginity and fertilisation of the females from embryo hatching. A series of egg batches were subsequently and independently collected. Each of these egg batches was incubated according to only one specific time point (i.e., representing individuals taken every hour from 1 to 12 h AEL).

### Embryo preparation for RNA isolation

Collected embryos were dechorionated in 3% sodium hypochlorite solution and washed in DEPC-treated water. Single embryos were individually transferred to a microcentrifuge tube (1.5 ml) before immediate grinding in 100 μl TRIzol reagent (Invitrogen, USA). The homogenates were kept at − 80 °C until the RNA isolation step.

### RNA and DNA isolation from adult samples

Genomic DNA was individually extracted according to Baruffi et al.^65^. Total RNA was isolated from 2-day old flies using TRIzol reagent according to the manufacturer′s instructions.

### RNA and DNA isolation from single embryos, larvae and pupae

Parallel RNA and genomic DNA extractions were carried out when using single embryos, larvae and pupae. Total RNA was isolated from each sample using TRIzol reagent as per the instructions from the manufacturer. During the RNA isolation, genomic DNA was extracted from the interphase and the organic phase of the TRIzol/chloroform mix^21^. The RNA precipitation required an additional 10 µg of RNase-free glycogen (Thermo Scientific, USA) for single embryos. The resuspended RNA solution was evaluated using NanoDrop spectrophotometer (Thermo Scientific, USA).

### Isolation of *tra-2* and *fru* orthologues

For *B. dorsalis* and *B. correcta tra-2* genes, the primers were designed from the conserved CDS of *Bactrocera* spp. and *C. capitata tra-2* genes (GenBank Acc. No. are presented in Supplementary Table S3) to achieve the full transcript of *Bdtra-2* and *Bctra-2* genes. One microgram of total RNA from adult males and females was converted to the first-strand cDNA using SMARTer RACE 5′/3′ Kit (Takara, USA) according to the protocol of the manufacturer. One-tenth of 5′- and 3′-RACE-Ready cDNA samples were subsequently used as templates for RT-PCR and 5′ and 3′ RACEs with gene-specific primers. Semi-nested RT-PCRs were performed to derive a more specific amplification according to SMARTer RACE 5′/3′ Kit. The negative controls were also performed by excluding reverse transcriptase. The *tra-2* gene structures were assembled based on PCR experiments using three pairs of PCR primers that were derived from *Bdtra-2* and *Bctra-2* full-transcripts and genomic DNA templates. The long *Bdtra-2* and *Bctra-2* cDNA and genomic DNA sequences were deposited to GenBank (Supplementary Table S3). The exon/intron junctions were deduced from these sequence alignments. A list of PCR primers in the *tra-2* studies can be found in Supplementary Table S4.

Total RNA from freshly emerged adult heads of both sexes was used in the study of *Bdfru* and *Bcfru* transcripts. Several gene-specific primer pairs were used to amplify *Bdfru* and *Bcfru* cDNA based on conserved sequences that encoded the BTB domain and four zinc-finger domains of the *fru* orthologues. These primers were designed from predicted *fru* transcripts in the transcriptome of many tephritid species and *M. domestica fru* transcripts (GenBank Acc. Nos. are presented in Supplementary Table S3). *Bdfru* and *Bcfru* common (C1 to C4) exon-encoded sequences were identified from the gene-specific RT-PCR using primers derived from the BTB coding sequence and its 3′ adjacent (Supplementary Fig. S2b and S2d). Four alternative 3′ exon-encoded cDNA sequences were amplified from the zinc-finger domain (ZnF A, ZnF B, ZnF C, and ZnF D) derived primers (Supplementary Fig. S2b and S2d). Four alternative 5′ exon sequences were isolated from 5′ RACE experiments using reverse primers derived from the homologous sequence of the C3 exon and the male-specific exon of *Mdfru* and *Ccfru*, respectively (Supplementary Fig. S2a and S2c). Similarly, the four alternative 3′ exon sequences were amplified from the 3′ RACE experiments using forward primers derived from the homologous sequence of the C4 exon of *Mdfru* (Supplementary Fig. S2a and S2c). The 5′- and 3′-RACE methods were done as previously described. The male-specific exon was identified by RT-PCR using a forward primer derived from the male-specific *Ccfru* sequence and gene-specific reverse primer derived from a homologous sequence of the C2 exon of *Mdfru*. The putative female-specific exon was amplified using specific primers derived from the available 1.9 kb genomic sequence adjacently downstream to the male-specific exon in the *B. dorsalis* genomic scaffold (GenBank Acc. No. NW_011876307). Intron/exon junctions of all isolated *Bdfru* transcripts were analysed by blasting them to the available *B. dorsalis* genomic scaffold (GenBank Acc. No. NW_011876307). The cDNA sequences of long *Bdfru* and *Bcfru* transcripts were deposited to GenBank (Supplementary Table S3). All primers used in the *fru* gene studies are described in Supplementary Table S4.

### RT-PCR analyses of sex-determining genes during early embryonic development and later stages

Sexual identification of single embryo, larval, and pupal samples were molecularly identified by genomic PCR using Y-specific primers which were designed based on sequences such as the *MoY* orthologue^14^. A *glyceraldehyde-3-phosphate dehydrogenase* (*gapdh*) gene-specific PCR was used to control false negative samples. Up to 300 ng of total RNA from single embryos, larvae, pupae, and adults were reverse transcribed into the first strand cDNA using ImProm-II Reverse Transcriptase (Promega, USA), following the manufacture′s instructions. One-tenth of the initial reverse transcription reaction was used as a template for RT-PCR using gene-specific primers. For each sample, the negative control of RT-PCR was performed by excluding the reverse transcriptase. The *gapdh* positive control system was also carried out in the RT-PCR. *Slam*, a zygotically expressed cellularisation gene, was used as an early developmental marker^21,55^. Therefore, the 2 to 3 h AEL embryos or older without detectable *slam* were discarded. Relative locations of gene-specific primers to each sex-determining gene were schematically plotted in Supplementary Table S5. These primer sequences are listed in Supplementary Table S6. All RT-PCR experiments gave the same electrophoretic banding patterns when they were repeated using cDNA templates of different individuals.

### *Bdtra* RNAi experiment

Approximately 1.1 kb dsRNA of the *Bdtra* gene was synthesised by in vitro transcription with T7 RNA polymerase using the MEGAscript Kit (Ambion, USA). Embryos were prepared from the *B. dorsalis* Salaya1 genetic sexing strain, whose sexual phenotypes can be separated by pupal colour dimorphism^64^. One μg/μl of *Bdtra* dsRNA was microinjected at the posterior end of precellular blastoderm embryos. The RNAi-treated individuals were reared until adult emergence. Wild-type male and female adults emerged from brown and white pupae, respectively. The white-pupae males were therefore categorised as pseudomales^24^. The genitalia and testes of the wild-type males and pseudomales were compared under a microscope. The sex-specific splicing pattern of *Bdtra*, *Bdtra-2*, *Bddsx*, and *Bdfru* were carried out using the previously described RT-PCR conditions in two pseudomales (randomly selected). The sex-specific splicing patterns of these genes were compared to the wild-type males and females as well as the RNAi-treated wild-type males.

### Molecular cloning and DNA sequencing of PCR amplification products

The specific amplicon was purified with the GF-1 AmbiClean Kit (Vivantis, Malaysia). The products were subsequently cloned into the pGEM-T Easy vector (Promega, USA) according to the manufacturer′s instructions. The recombinant plasmids were transformed into *E. coli* (DH5α) competent cells and screened for positive colonies. Plasmids were extracted using the GF-1 Plasmid DNA Extraction Kit (Vivantis, Malaysia). All sequencing reactions were performed on both strands using the sequencing service from Macrogen Inc., Seoul, Korea.

### DNA sequence analysis

Query DNA sequences were searched for homology using blast against the NCBI database for gene identification. Genomic DNA and cDNA sequences were aligned using the ClustalX2 program^66^ and Unipro UGENE Version 1.14.0^67^. For sequence analysis of *Bdfru*, the genomic scaffold (GenBank Acc. No. NW_011876307) was used to predict the gene structure. GenBank accession numbers of the references and the newly isolated sequences are in Supplementary Table S3.

### Morphological development of early embryos

The morphology of live embryos was observed during early development (0 to 9 h AEL) under differential interference contrast using an inverted confocal laser scanning microscope (Olympus FV1000). The collection of developing embryos, at each time point, was as previously described. The live embryos were gently dechorionated using 3% sodium hypochlorite solution for 5 min and then transferred in a drop of 1X phosphate buffer saline to a glass slide before observation and photography.

### Ethics statement

This manuscript is a part of a research project which has been approved by Mahidol University-Institute Animal Care and Use Committee (no. MU-IACUC 2018/007). This research also complies with Biosafety Guildeline for Modern Biotechnology from Institutional Biosafety Committee under Pathogens and Animal Toxins Act, B.E. 2558 (2015); (Exempt2019-003).

## Supplementary information

Supplementary Figures.

Supplementary Tables.

## Data Availability

All relevant data are included in the manuscript and the Supplementary Information files.
