# Supplementary material for: The transformer-2 and fruitless characterisation with developmental expression profiles of sex-determining genes in Bactrocera dorsalis and B. correcta
Source: Sci Rep. 2020 Oct 21;10:17938. doi: 10.1038/s41598-020-74856-6 (PMC7578103; doi:10.1038/s41598-020-74856-6)
Supplement: Supplementary file 1 — Supplementary Figures. [file 41598_2020_74856_MOESM1_ESM.pdf]

Title: The *transformer-2* and *fruitless* characterisation with developmental expression profiles of sex-determining genes in *Bactrocera dorsalis* and *B. correcta*

Kamoltip Laohakieat[1], Siriwan Isasawin[1], Sujinda Thanaphum[1,*]

[1] Regional R&D Training Center for Insect Biotechnology (RCIB), Department of Biotechnology, Faculty of Science, Mahidol University, Rama VI Road, Bangkok 10400, Thailand

[*]corresponding author: sujinda.tha@mahidol.ac.th

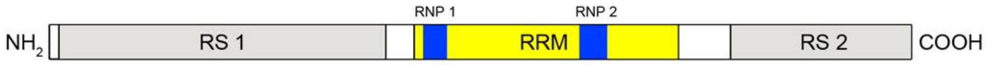


```
BdTRA-2     MS--PRSRSR-SISPRRSYTKSPVRRDNGR----RRHSREKVYSSRSRSASRQPPSPPPL    53
BcTRA-2     MS--PRSRSR-SISPRRSYTKSPVRRNNGR----RRHSREKVYSSRSRSATRQPPSPPPL    53
BjTRA-2     MS--PRSRSR-SISPRRSYTKSPVRRGNGR----RRHSKEKMYSSRSRSASRQPPSPPPL    53
BtTRA-2     MS--PRSRSR-SISPRRSYTKSPVRRGNGRRFLNRRHSKEKVYSSRSRSASRQPPSPPPL    57
BoTRA-2     MS--PRSRSR-SLSPRRSYTKSPVRRGNGR----RRHSREKFYSSRSRSASRQPPSPPPL    53
ZcuTRA-2    MS--PRSRSR-SISPRRSYTKSPVRRGNGR----RRHSREKVYDSRSRSVSRQPPSPPPL    53
CcTRA-2     MS--PRSRSR-SISARRSYTKSPARRSNGR----RRHSREKVYKSRSRSISRHPPSPPPP    53
AsTRA-2     MS--PRTRSR-SISPRRSYSKSPARRSNGR----RRHSREKVYNSRSRSASRHPPSPPPL    53
AfTRA-2     MS--PRTRSR-SISPRRSYSKSPARRSNGR----RRHSREKVYNSRSRSASRHPPSPPPL    53
AoTRA-2     MS--PRTRSR-SISPRRSYSKSPARRSNGR----RRHSREKVYNSRSRSASRHPPSPPPL    53
DmTRA-2     MDREPLSSGRLHCSARYKHKRSASSSSAGT----TSSGHKDRRSDYDYCGSRRHQ-----    51
            *.  * : .*   *.* .:.:*.   . *        .::.  .. . . :*:

                                                                RNP1
BdTRA-2     PSGRGG-RYSEASQSSSTSLSPRHGRRISRSRSRSTYEKRRGNREKPVQNRCIGVFGLSV    112
BcTRA-2     PSGRGG-RYSEASQSSSTSLSPRHGRRISRSRSRSAYEKRRGNREKPVQNRCIGVFGLSV    112
BjTRA-2     PSGRGG-RYSEASQSSSTSLSPRHGRRISRSRSRSTYEKRRGNREKPVQNRCIGVFGLSV    112
BtTRA-2     PSGRGG-RYSEASQSSSTSLSPRHGRRISRSRSRSTYEKRRGNREKPVQNRCIGVFGLSV    116
BoTRA-2     PSGRGG-RYSEASQSSSTSLSPRHGRRITRSRSRSTHENRRGNREKPVQNRCIGVFGLSV    112
ZcuTRA-2    PTGRGG-RYSEASQSSSTSLSPRHGRRVSRSRSRSTYEKRRGNREKPVQNRCIGVFGLSV    112
CcTRA-2     PTGRGGVRCSDASQSSSTSLSPRQGRRMSRSRSRSPYDKRRGNREKPVQNRCIGVFGLSV    113
AsTRA-2     PAGRAG-RYSDASKSSSTSLSPRHGRRVSRSRTRSPYDKRRANREKPVQNRCIGVFGLSV    112
AfTRA-2     PAGRAG-RYSDASKSSSTSLSPRHGRRVSRSRTRSPYDKRRANREKPVQNRCIGVFGLSV    112
AoTRA-2     PAGRAG-RYSDASKSSSTPLSPRHGRRVSRSRSRSPYDKRRANREKPVQNRCIGVFGLSV    112
DmTRA-2     ---RSSSRRRSRSRSSSESPPPEPRHRSGRS-SRD-RERMHKSREHPQASRCIGVFGLNT    106
               *.. *  . *:*** . .*.  :*  ** :*.  :. : .**:*  .********..

                                           RNP2
BdTRA-2     YTTQQKIRDIFSRFGPIERIQVVIDAQTGRSRGFCFIYYEDIADAKAAKDACSGMEIDDR    172
BcTRA-2     YTTQQKIRDIFSRFGPIERIQVVIDAQTGRSRGFCFIYYEDIADAKAAKDACSGMEIDDR    172
BjTRA-2     YTTQQKIRDIFSRFGPIERIQVVIDAQTGRSRGFCFIYYEDIADAKAAKDACSGMEIDDR    172
BtTRA-2     YTTQQKIRDIFSRFGPIERIQVVIDAQTGRSRGFCFIYYEDIADAKAAKDACSGMEIDDR    176
BoTRA-2     YTTQQKIRDIFSRFGPIERIQVVIDAQTGRSRGFCFIYYEDIADAKAAKDACSGMEIDDR    172
ZcuTRA-2    YTTQQKIRDIFSRFGPIERIQVVIDARTGRSRGFCFIYYEDIADAKAAKDACSGMEIDDR    172
CcTRA-2     YTTQQKIRDIFSRFGPIERIQVVIDAQTGRSRGFCFIYYDDIADAKAAKDACSGMEIDDR    173
AsTRA-2     YTTQQKIRDIFSRFGPIERIQVVIDAQTGRSRGFCFIYYQDIADAKAAKDACSGMEINDR    172
AfTRA-2     YTTQQKIRDIFSRFGPIERIQVVIDAQTGRSRGFCFIYYQDIADAKAAKDACSGMEIDDR    172
AoTRA-2     YTTQQKIRDIFSRFGPIERIQVVIDAQTGRSRGFCFIYYQDIADAKAAKDACSGTEIDDR    172
DmTRA-2     NTSQHKVRELFNKYGPIERIQMVIDAQTQRSRGFCFIYFEKLSDARAAKDSCSGIEVDGR    166
             *:*:*:*::*.::*******:****:* *********::.::**:****:*** *::.*

BdTRA-2     RIRVDYSTTQRPHTPTPGVYMGRQTRREREHSDRYRDDYRPRR-RSGSPYHSRN--NNYR    229
BcTRA-2     RIRVDYSTTQRPHTPTPGVYMGRQTRREREHNDRYRDDYRPRR-RSGSPYHSRN--NNYR    229
BjTRA-2     RIRVDYSTTQRPHTPTPGVYMGRQTRREREHNDRYRDDYRPRR-RSGSPYHSRN--NNYR    229
BtTRA-2     RIRVDYSTTQRPHTPTPGVYMGRQTRREREHNDRYRDDYRPRR-RSGSPYHSRN--NNYR    233
BoTRA-2     RIRVDYSTTQRPHTPTPGVYMGRQTRREREHNDRYRDDYRPRR-RSGSPYHNRS--NNYR    229
ZcuTRA-2    RIRVDYSTTQRPHTPTPGVYMGRHTRREREHNDRYRDDYRSRR-RSGSPYHSR---NNYR    228
CcTRA-2     RIRVDYSTTQRPHTPTPGVYMGRHTRREREYNDRYRDDYRPRR-RSGSPFKNR---NNYR    229
AsTRA-2     RIRVDYSTTQRPNTPTPGVYMGRYTRRERDH-DRYRDDYRSRR-RSVTPHNSR---NSYR    227
AfTRA-2     RIRVDYSTTQRPHTPTPGVYMGRYTRRERDH-DRYRDDYRSRR-RSVTPHNSR---NSYR    227
AoTRA-2     RIRVDYSTTQRPHTPTPGVYMGRYTRRERDH-DRYRDDYRSRR-RSVTPHNSR---NSYR    227
DmTRA-2     RIRVDFSITQRAHTPTPGVYLGRQPRGKAPRSFSPRRGRRVYHDRSASPYDNYRDRYDYR    226
            *****:* ***.:*******:** .* :       * . *  : ** :*...     .**

BdTRA-2     SDR----------RRRYERSR--SRSYSP--RR--ARY    251
BcTRA-2     SDR----------RRRYERSR--SRSYSP--RR--ARY    251
BjTRA-2     SDR----------RRRYERSR--SRSYSP--RR--ARY    251
BtTRA-2     SDR----------RRRYERSR--SRSYSP--RR--ARY    255
BoTRA-2     SDR----------SRRYERSR--SRSYSP--RR--ARY    251
ZcuTRA-2    SER----------RRRYERSR--SRSYSP--RR--ARY    250
CcTRA-2     NDR----------RRRYDRSR--SRSYSP--RR--ARY    251
AsTRA-2     GDR----------RRRYDRSR--SRSYSP--RR--ARY    249
AfTRA-2     GDR----------RRRYDRSR--SRSYSP--RR--TRY    249
AoTRA-2     GDR----------RRRYDRSR--SRSYSP--RR--TRY    249
DmTRA-2     NDRYDRNLRRSPSRNRYTRNRSYSRSRSPQLRRTSSRY    264
            .:*           .** *.*  *** **  **  :*
```

Supplementary Figure S1. Predicted schematic of TRA-2 polypeptides and amino acid sequence alignment from tephritid fruit flies and *D. melanogaster*. The same colour represents protein domain or region of the upper schematic and amino acid sequence alignment. The RNA Recognition Motif (RRM) domain is highlighted with yellow, and the flanking regions are arginine/serine (RS) rich regions (grey shaded). The ribonucleoprotein 1 (RNP1) and ribonucleoprotein 2 (RNP2) are in the blue boxes. The characteristic features of RRM domains are highly conserved across the fruit fly species. The GenBank Acc. Nos. of TRA-2 amino acid sequences are in Supplementary Table S3.

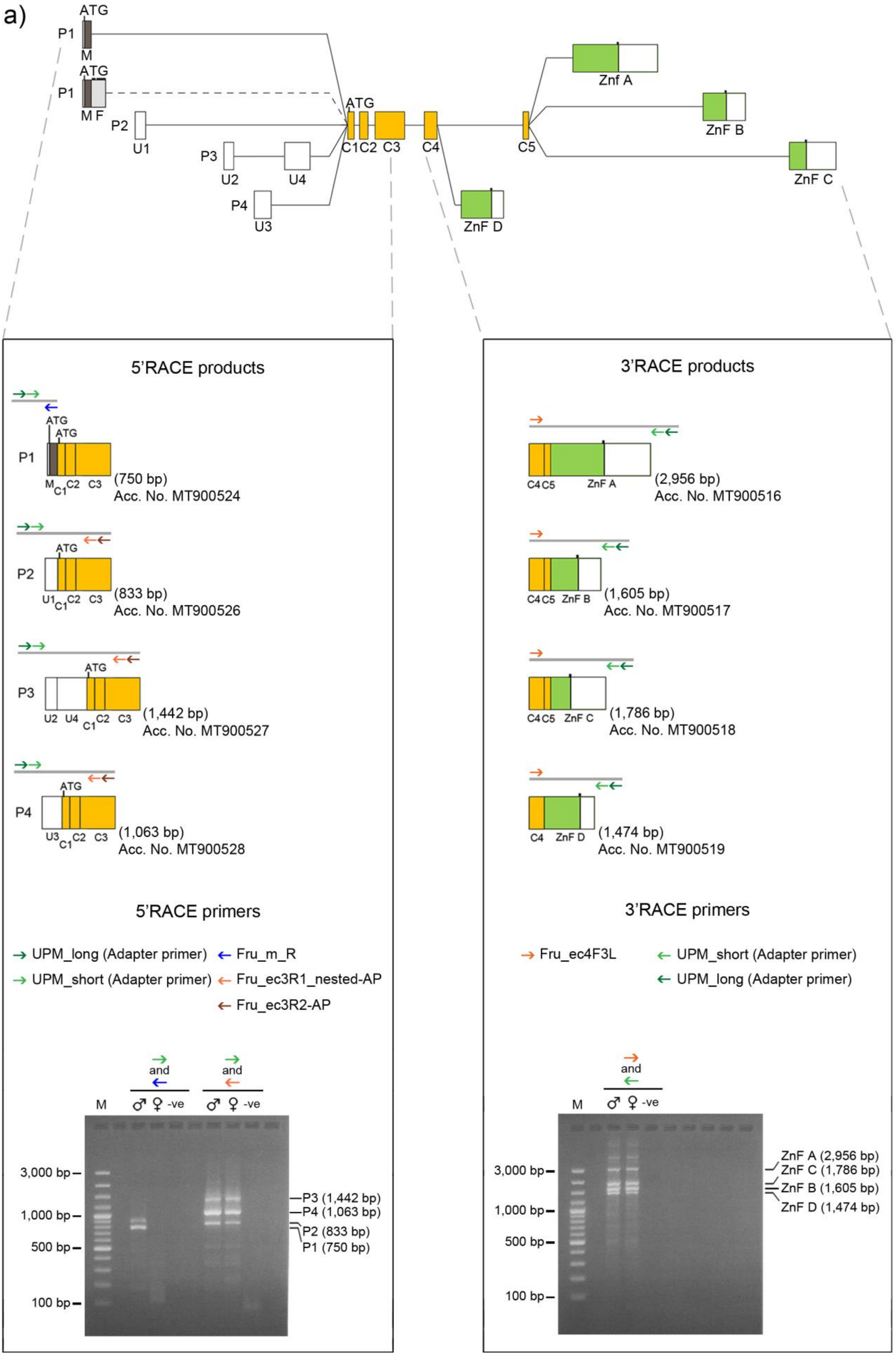
a)
P1
ATG
M
P1
ATG
M F
P2
U1
P3
U2
U4
P4
U3
ATG
C1 C2 C3
C4
C5
Znf A
ZnF B
ZnF C
ZnF D
5'RACE products
P1
ATG
ATG
M C1 C2 C3
(750 bp)
Acc. No. MT900524
P2
ATG
U1 C1 C2 C3
(833 bp)
Acc. No. MT900526
P3
ATG
U2 U4 C1 C2 C3
(1,442 bp)
Acc. No. MT900527
P4
ATG
U3 C1 C2 C3
(1,063 bp)
Acc. No. MT900528
5'RACE primers
UPM_long (Adapter primer)
UPM_short (Adapter primer)
Fru_m_R
Fru_ec3R1_nested-AP
Fru_ec3R2-AP
and
and
M
♂ ♀ -ve
♂ ♀ -ve
3,000 bp
1,000 bp
500 bp
100 bp
P3 (1,442 bp)
P4 (1,063 bp)
P2 (833 bp)
P1 (750 bp)
3'RACE products
C4 C5 ZnF A
(2,956 bp)
Acc. No. MT900516
C4 C5 ZnF B
(1,605 bp)
Acc. No. MT900517
C4 C5 ZnF C
(1,786 bp)
Acc. No. MT900518
C4 ZnF D
(1,474 bp)
Acc. No. MT900519
3'RACE primers
Fru_ec4F3L
UPM_short (Adapter primer)
UPM_long (Adapter primer)
and
M
♂ ♀ -ve
3,000 bp
1,000 bp
500 bp
100 bp
ZnF A (2,956 bp)
ZnF C (1,786 bp)
ZnF B (1,605 bp)
ZnF D (1,474 bp)

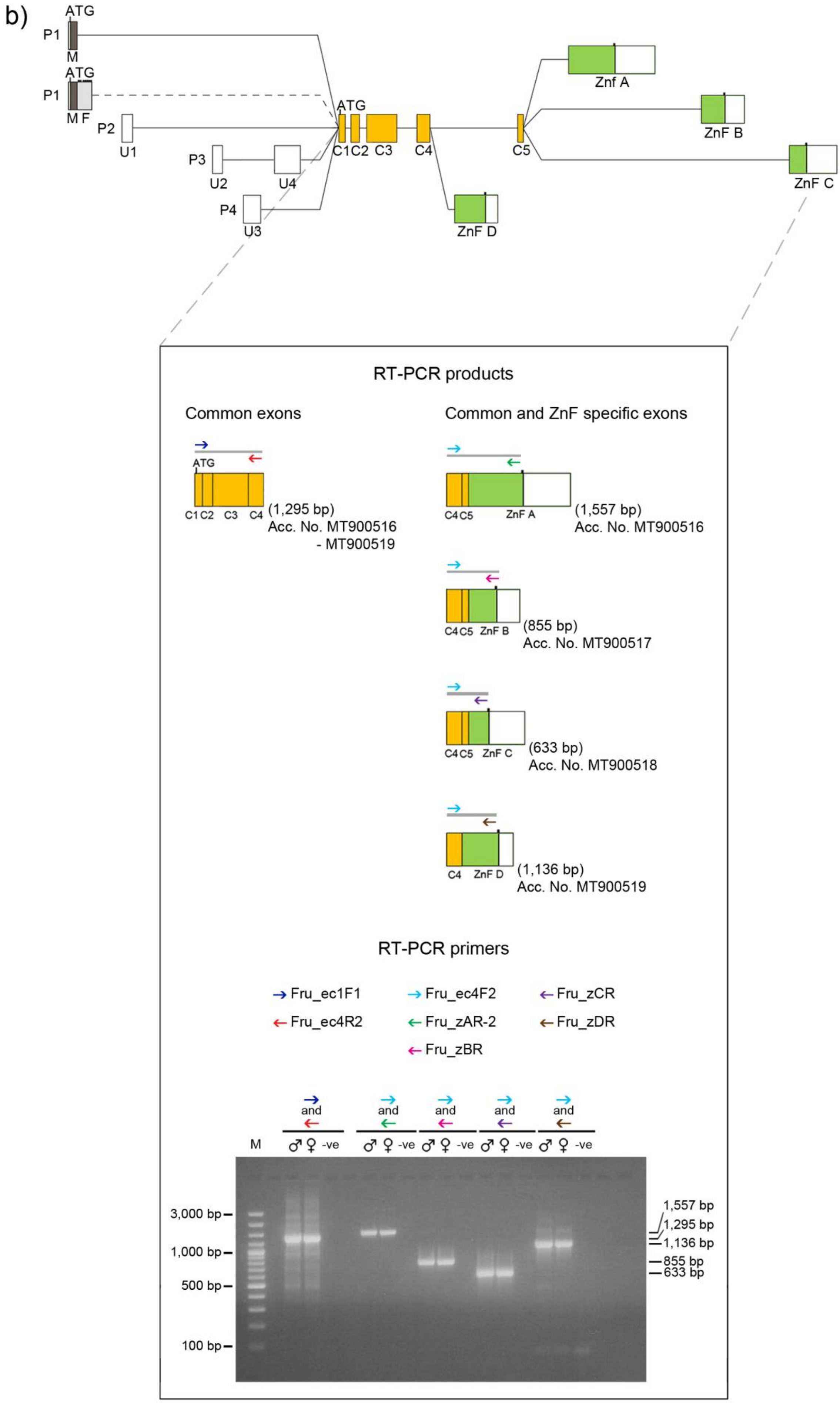
b)
ATG
P1
M
ATG
P1
M F
P2
U1
P3
U2
U4
P4
U3
ATG
C1C2 C3
C4
C5
Znf A
ZnF B
ZnF C
ZnF D
RT-PCR products
Common exons
ATG
C1 C2 C3 C4
(1,295 bp)
Acc. No. MT900516 - MT900519
Common and ZnF specific exons
C4 C5 ZnF A
(1,557 bp)
Acc. No. MT900516
C4 C5 ZnF B
(855 bp)
Acc. No. MT900517
C4 C5 ZnF C
(633 bp)
Acc. No. MT900518
C4 ZnF D
(1,136 bp)
Acc. No. MT900519
RT-PCR primers
Fru_ec1F1
Fru_ec4R2
Fru_ec4F2
Fru_zAR-2
Fru_zBR
Fru_zCR
Fru_zDR
and
M
♂ ♀ -ve
3,000 bp
1,000 bp
500 bp
100 bp
1,557 bp
1,295 bp
1,136 bp
855 bp
633 bp

c)

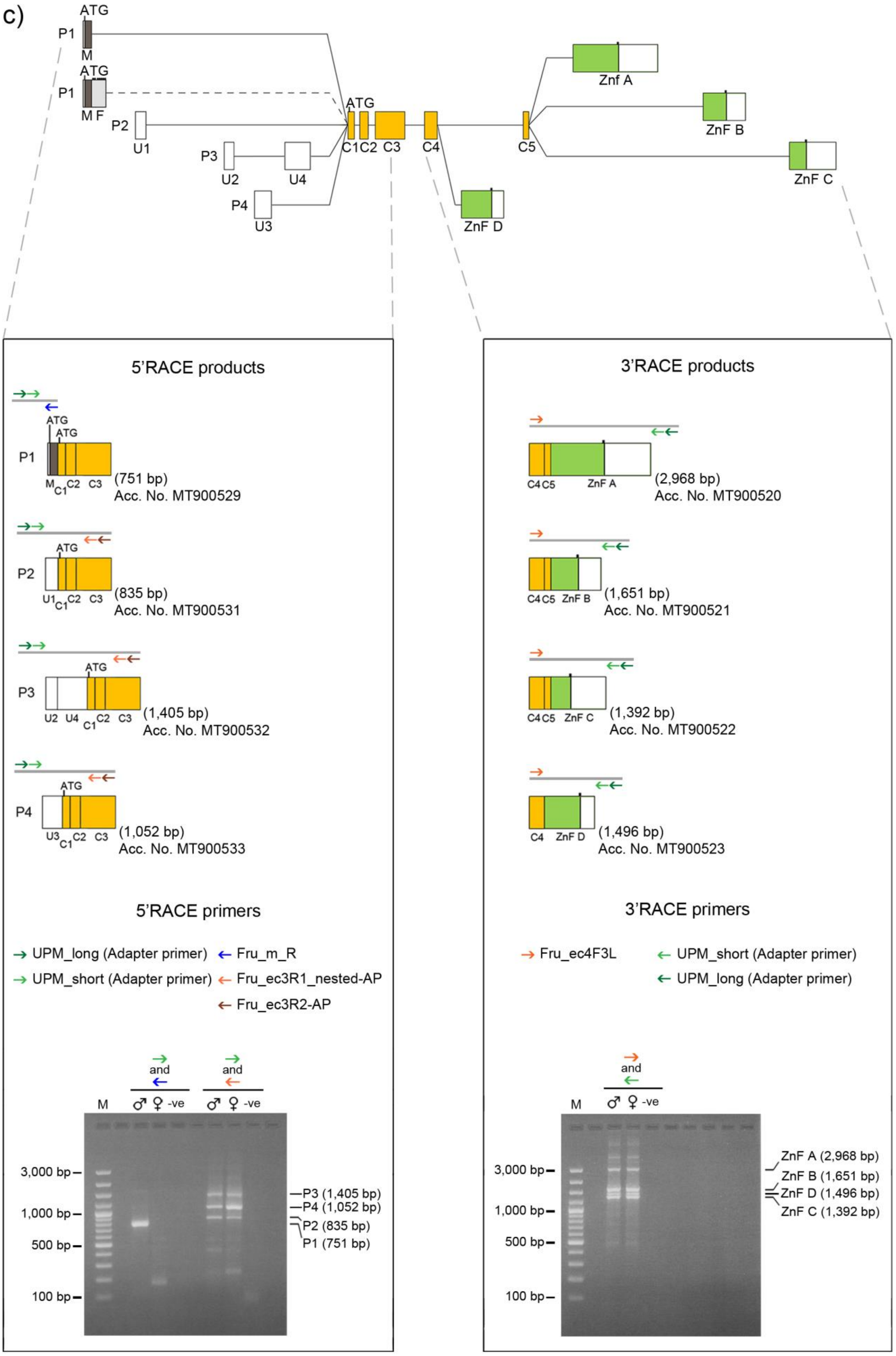

ATG
P1
M
ATG
P1
M F
P2
U1
ATG
C1 C2 C3
C4
C5
P3
U2
U4
P4
U3
Znf A
ZnF B
ZnF C
ZnF D
5'RACE products
P1
(751 bp)
Acc. No. MT900529
P2
(835 bp)
Acc. No. MT900531
P3
(1,405 bp)
Acc. No. MT900532
P4
(1,052 bp)
Acc. No. MT900533
5'RACE primers
UPM_long (Adapter primer)
UPM_short (Adapter primer)
Fru_m_R
Fru_ec3R1_nested-AP
Fru_ec3R2-AP
3'RACE products
(2,968 bp)
Acc. No. MT900520
(1,651 bp)
Acc. No. MT900521
(1,392 bp)
Acc. No. MT900522
(1,496 bp)
Acc. No. MT900523
3'RACE primers
Fru_ec4F3L
UPM_short (Adapter primer)
UPM_long (Adapter primer)
and
M
♂ ♀ -ve
3,000 bp
1,000 bp
500 bp
100 bp
P3 (1,405 bp)
P4 (1,052 bp)
P2 (835 bp)
P1 (751 bp)
ZnF A (2,968 bp)
ZnF B (1,651 bp)
ZnF D (1,496 bp)
ZnF C (1,392 bp)

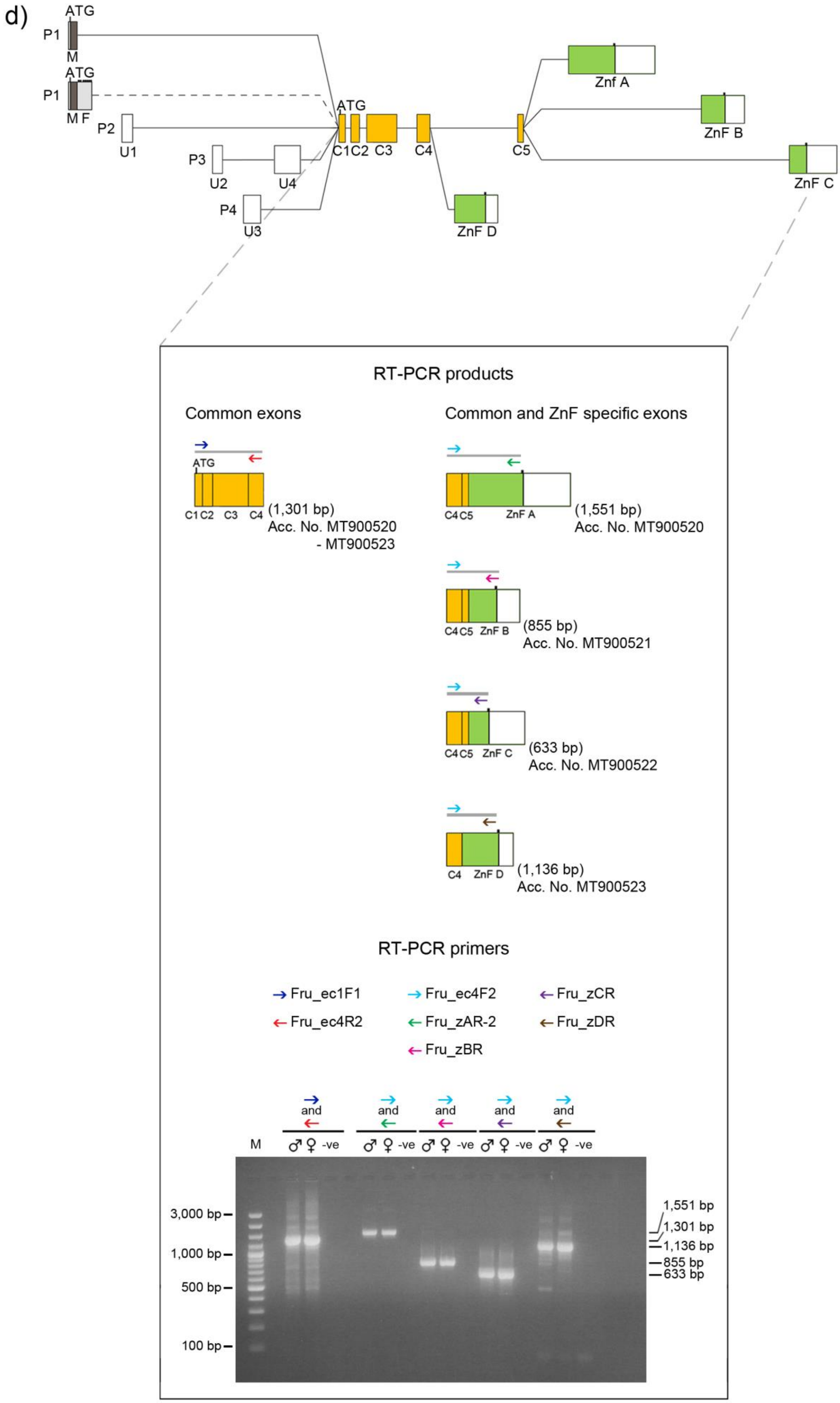
d)
ATG
P1
M
ATG
P1
M F
P2
U1
P3
U2
U4
P4
U3
ATG
C1C2 C3
C4
C5
Znf A
ZnF B
ZnF C
ZnF D
RT-PCR products
Common exons
ATG
C1 C2 C3 C4
(1,301 bp)
Acc. No. MT900520 - MT900523
Common and ZnF specific exons
C4 C5 ZnF A
(1,551 bp)
Acc. No. MT900520
C4 C5 ZnF B
(855 bp)
Acc. No. MT900521
C4 C5 ZnF C
(633 bp)
Acc. No. MT900522
C4 ZnF D
(1,136 bp)
Acc. No. MT900523
RT-PCR primers
Fru_ec1F1
Fru_ec4R2
Fru_ec4F2
Fru_zAR-2
Fru_zBR
Fru_zCR
Fru_zDR
and
and
and
and
and
M
♂ ♀ -ve
♂ ♀ -ve
♂ ♀ -ve
♂ ♀ -ve
♂ ♀ -ve
3,000 bp
1,000 bp
500 bp
100 bp
1,551 bp
1,301 bp
1,136 bp
855 bp
633 bp

Supplementary Figure S2. Transcriptional-organisation analyses of the *Bdfru* and *Bcfru* genes in *B. dorsalis* ((a) and (b)) and *B. correcta* ((c) and (d)), respectively, for Figure 2a. The schematic drawings show various possibilities of the *fru* transcripts. All 5′-, 3′-RACEs and RT-PCR products (with sizes and GenBank Acc. Nos.); the respective RACE and RT-PCR primer names and their locations; and the gel electrophoretic images of the PCR products are illustrated. More details on the transcriptional analyses of sex-specific exons (P1) are in Supplementary Figure S5. M is the 100 bp DNA ladder plus, ranging from 100 to 3,000 bp. Lane -ve was used as a negative control for each experiment. The primers used in this experiment are shown in Supplementary Table S4. The cDNA templates were prepared from freshly emerged adult heads of both sexes.

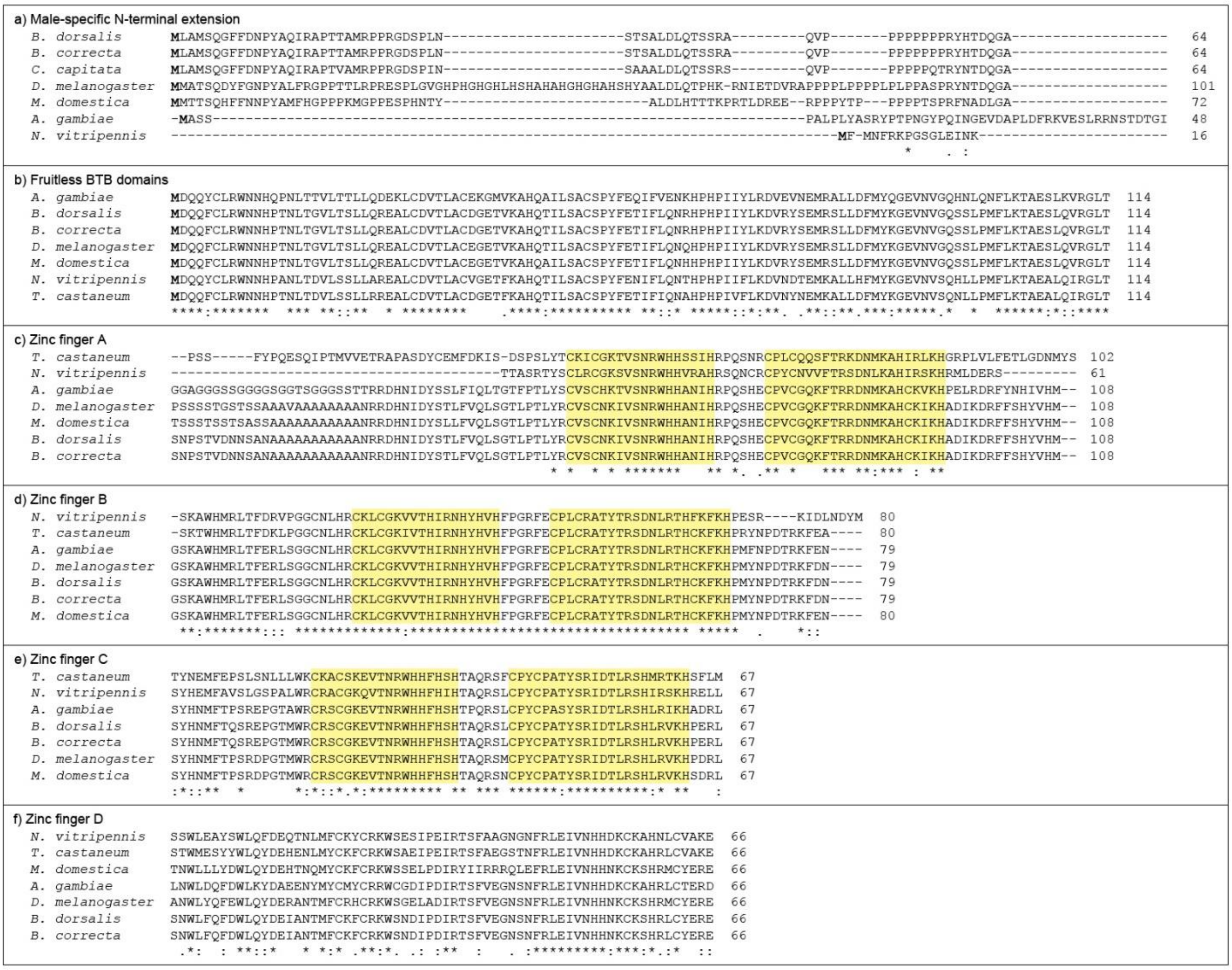

Supplementary Figure S3. Amino acid sequence alignments of FRU domains in different dipterans, *Tribolium*, and *Nasonia*. The alignments show (a) male-specific N-terminal extension of male-specific FRU (FRU^{M}) derived from the P1 promoter and (b) the BTB domains. The putative start codon is signified by a bold M. The alignment of the BTB domain shows a highly conserved pattern among these species. The alignments of partial amino acid sequences of zinc-fingers A, B, C, and D FRU show the conserved region of each zinc-finger encoding domain (c to f, respectively). The zinc-finger domains were predicted from previous reports[1,2] and the zinc-finger domain predictor[3,4] and highlighted with yellow. Asterisks (*), colons (:), and dots (.) indicate the identical, highly similar, and weakly similar amino acids, respectively. GenBank Acc. Nos. of FRU amino acid sequences are presented in Supplementary Table S3.

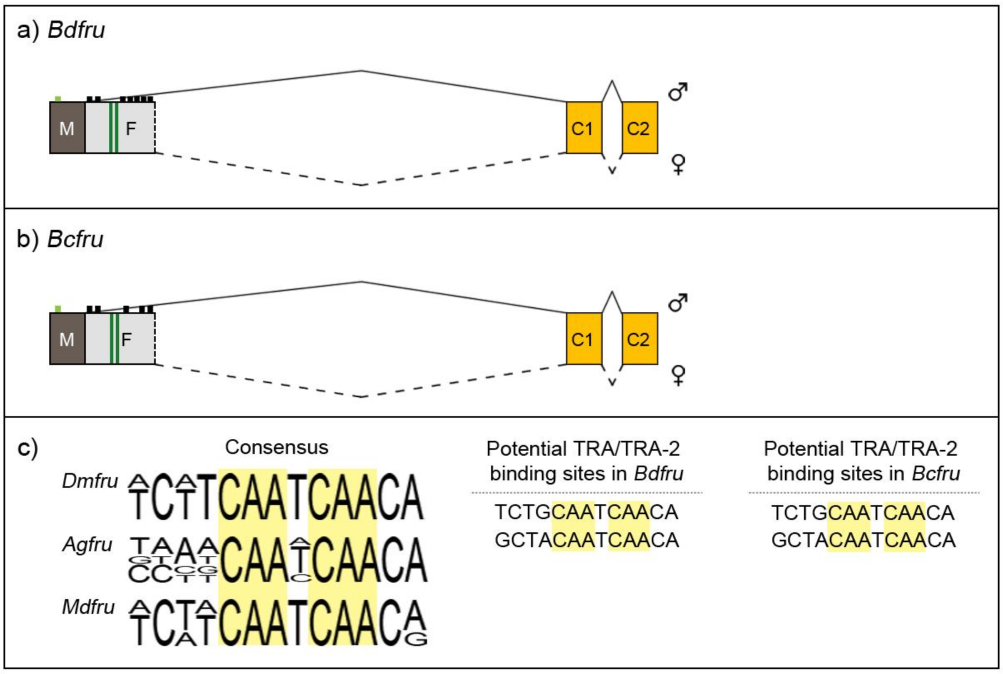

Supplementary Figure S4. Identification of the putative female-specific exons in *Bdfru* (a) and *Bcfru* (b) genes. The putative female-specific (F) exon as shown in the grey box is adjacent to the male-specific (M) exon. Small green and black dots mark the potential start codon and in-frame stop codons, respectively. The green bars located on the F exon represent the putative TRA/TRA-2 binding sites. Boxes and lines represent exons and alternative splicing patterns, respectively. The male-specifically spliced transcript is indicated with a black line. The connection between the F and common exons (dashed line) is still hypothetical because the RT-PCR validation was not positive. (c) Predicted TRA/TRA-2 binding sites found in *Bdfru* and *Bcfru* F exons are comparable to the consensus sequence of TRA/TRA-2 binding sites of *fru* genes from the other insects such as *D. melanogaster* (*Dmfru*)[1,2,5], *Ae. gambiae* (*Agfru*)[6], and *M. domestica* (*Mdfru*)[7]. The yellow highlighted regions represent the conserved "CAA" repeats among all sequences.

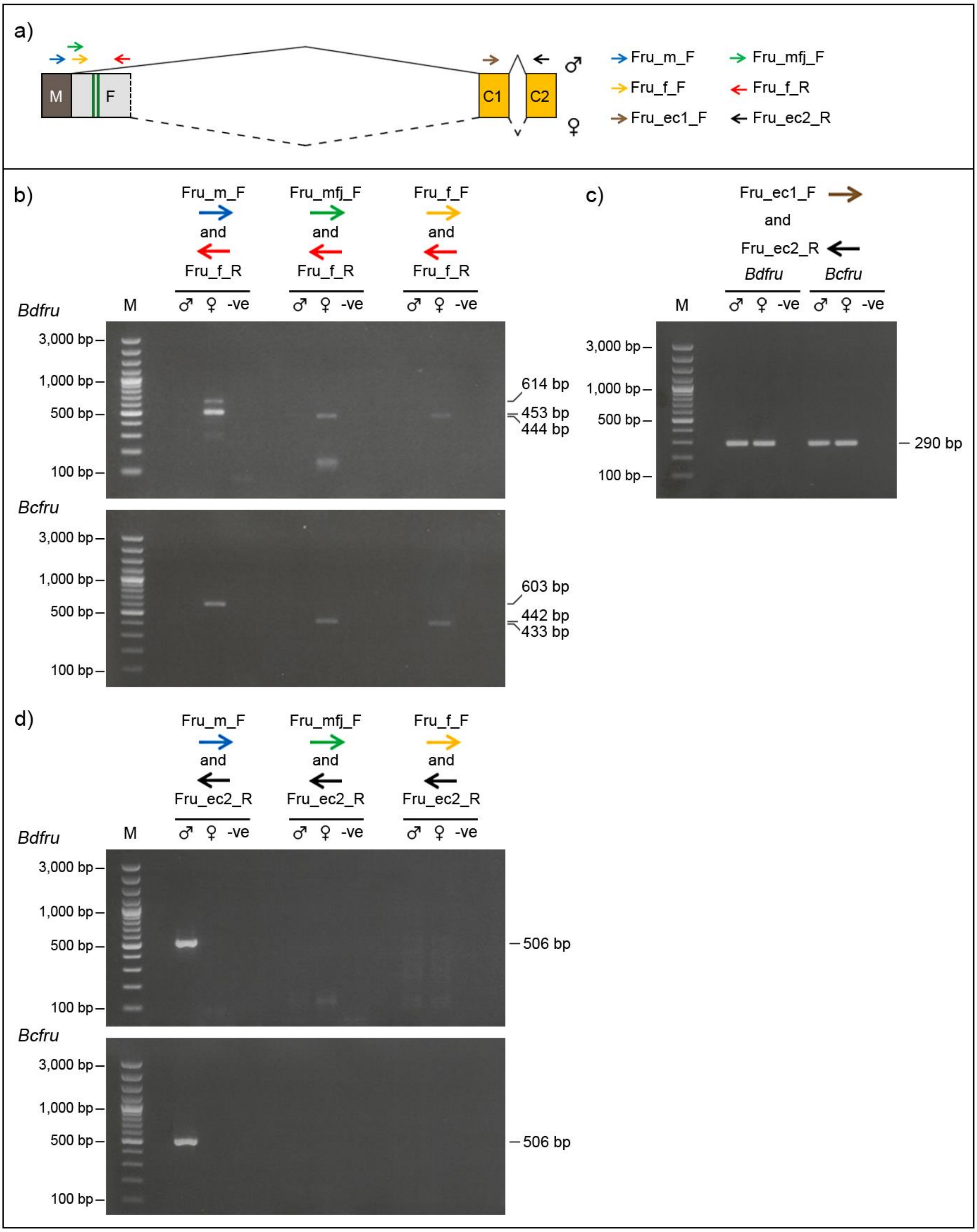

Supplementary Figure S5. RT-PCR analysis of the putative female-specific exons in *Bdfru* and *Bcfru* transcripts. (a) The primers used in the following amplifications are indicated by arrows with different colours. (b) An RT-PCR experiment was performed to identify the presence of the putative

F exon and the connection to the M exon. (c) An RT-PCR assay of common C1 and C2 exons was used as a positive control. (d) RT-PCR was carried out to identify the connection between the putative F exon and the BTB encoding exons. Negative control (-ve) of each RT-PCR set was performed without a cDNA template. The primers used in this experiment are shown in Supplementary Table S4. M is the 100 bp DNA ladder plus. The full-length gels are presented in Supplementary Figure S11.

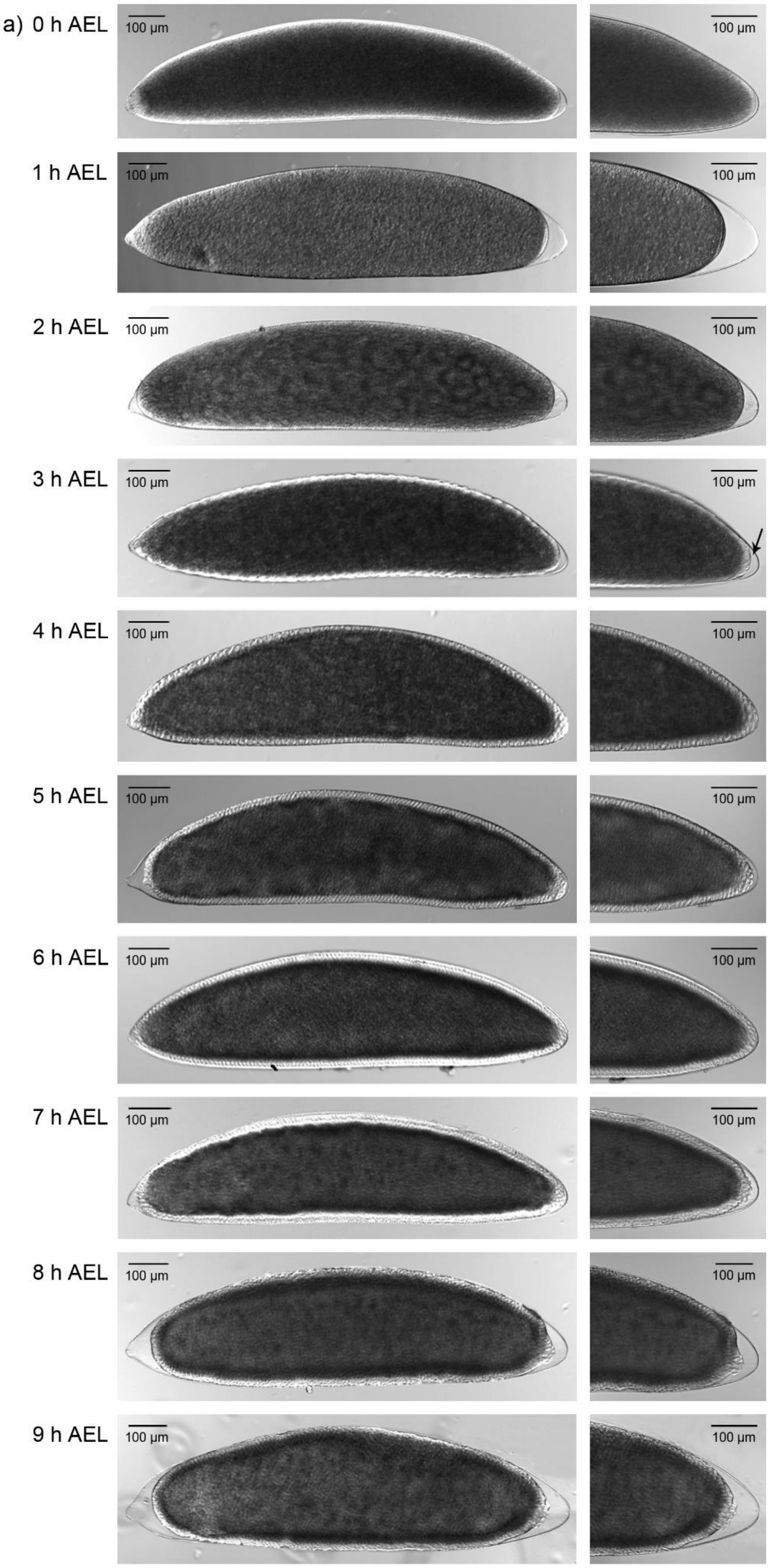
a)
0 h AEL
1 h AEL
2 h AEL
3 h AEL
4 h AEL
5 h AEL
6 h AEL
7 h AEL
8 h AEL
9 h AEL
100 µm

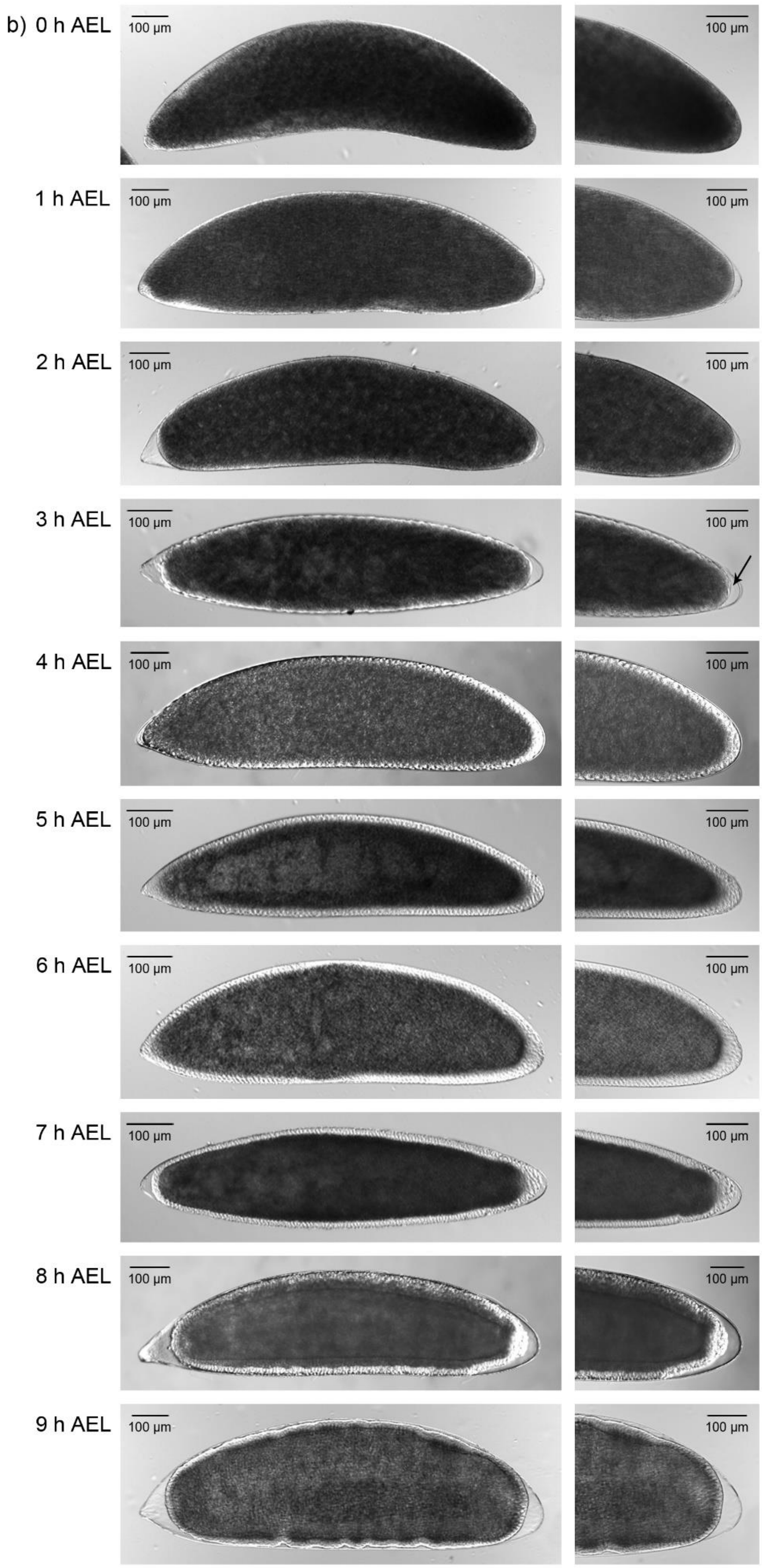
b) 0 h AEL
100 µm
100 µm
1 h AEL
100 µm
100 µm
2 h AEL
100 µm
100 µm
3 h AEL
100 µm
100 µm
4 h AEL
100 µm
100 µm
5 h AEL
100 µm
100 µm
6 h AEL
100 µm
100 µm
7 h AEL
100 µm
100 µm
8 h AEL
100 µm
100 µm
9 h AEL
100 µm
100 µm

Supplementary Figure S6. Early embryonic development of (a) *B. dorsalis* and (b) *B. correcta* at 25ºC. Embryos were fixed at one hour intervals from 0 to 9 h AEL. At the posterior pole, the arrow indicates the pole cells appearing at about 3 h AEL. The embryos show different developmental stages, such as before cellularisation (0 to 6 h AEL), during cellularisation (7 to 8 h AEL), and gastrulation (8 to 9 h AEL).

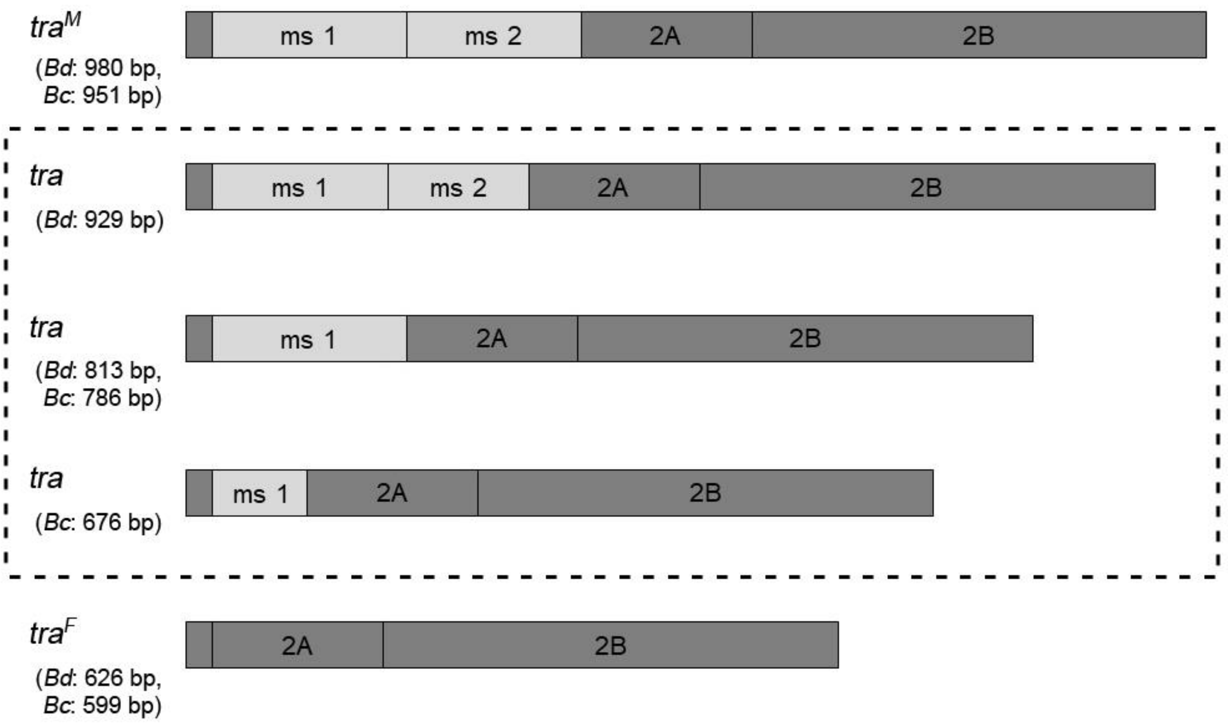

Supplementary Figure S7. Schematic of sex-specifically spliced and intermediate *Bdtra* and *Bctra* transcripts detected in 5 to 8 h AEL embryos. The dark gray boxes represent the common exons (1B, 2A, and 2B). The light grey boxes indicate male-specific exons (ms 1 and ms 2). Male- and female- specifically spliced transcripts are represented as tra^M and tra^F, respectively. The dashed rectangle contains *tra* intermediate transcripts which are partially spliced. Amplicon sizes of *Bdtra* and *Bctra* transcripts are in parentheses.

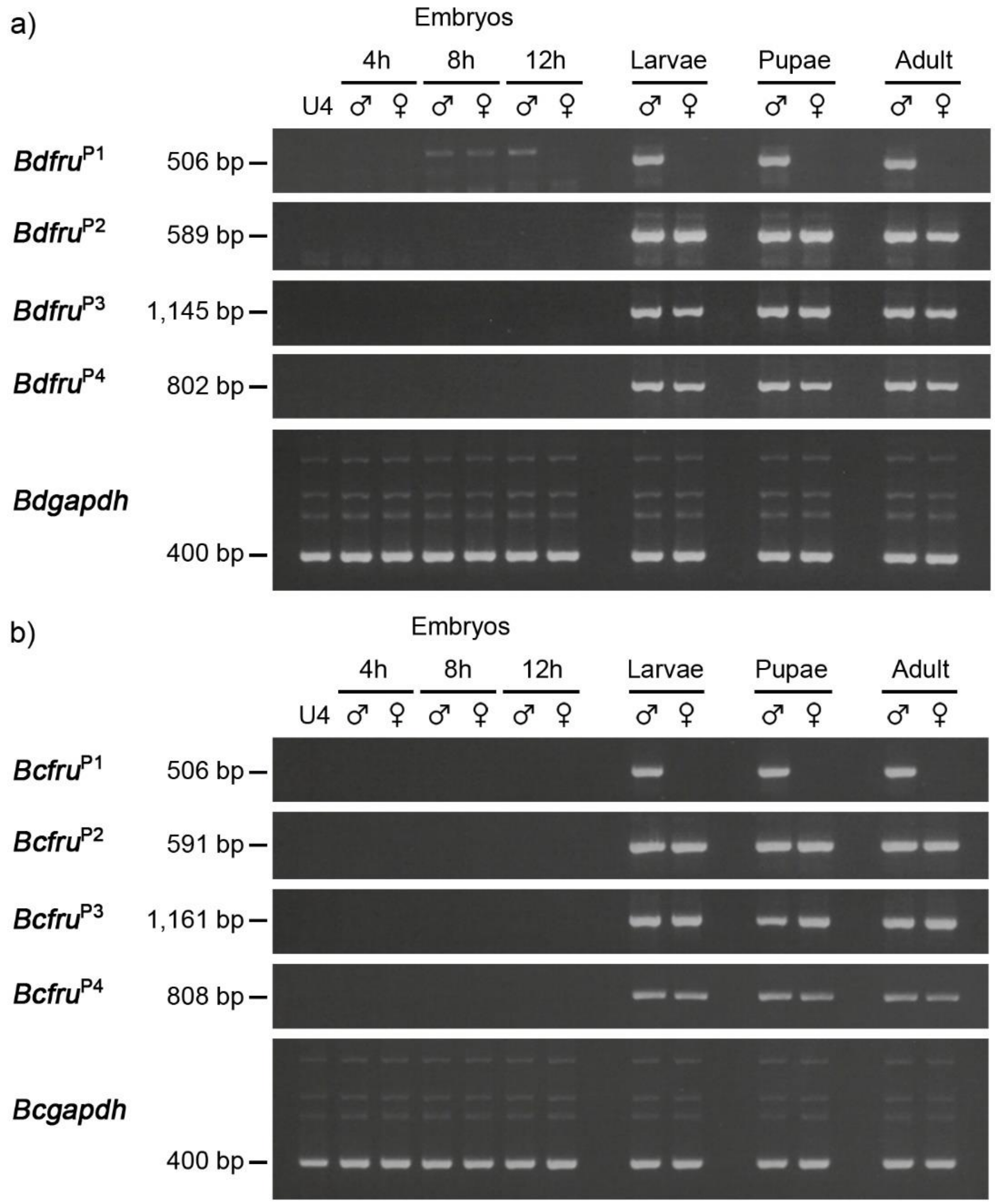

Supplementary Figure S8. Expression analysis of the P1 to P4 transcripts during developmental stages of (a) *Bdfru* and (b) *Bcfru* genes. RT-PCRs were performed using the forward primer located on the M or U exons derived from different transcript classes (P1 to P4) and the reverse primer located on the common C2 exon as shown in the Figure 2. *Bdgapdh* was used as a positive control for the experiment. The cDNA samples were considered when the reaction without RTase produced no amplicons. The primers used in this experiment are shown in Supplementary Table S4. The full-length gels are presented in Supplementary Figure S12.

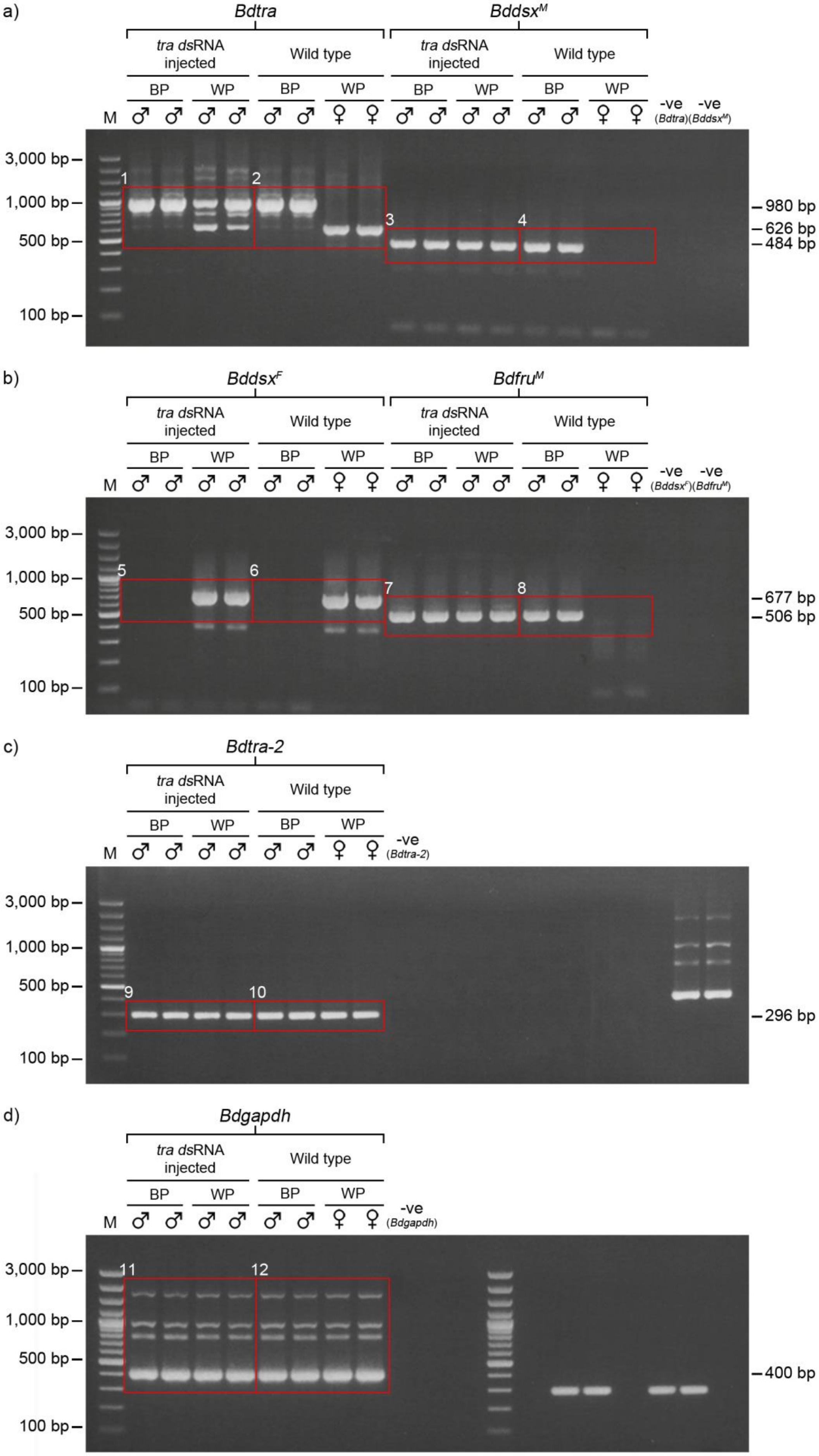
a)
Bdtra
BddsxM
tra dsRNA injected
Wild type
BP
WP
M
-ve (Bdtra)(BddsxM)
3,000 bp
1,000 bp
500 bp
100 bp
980 bp
626 bp
484 bp
1
2
3
4
b)
BddsxF
BdfruM
tra dsRNA injected
Wild type
BP
WP
M
-ve (BddsxF)(BdfruM)
3,000 bp
1,000 bp
500 bp
100 bp
677 bp
506 bp
5
6
7
8
c)
Bdtra-2
tra dsRNA injected
Wild type
BP
WP
M
-ve (Bdtra-2)
3,000 bp
1,000 bp
500 bp
100 bp
296 bp
9
10
d)
Bdgapdh
tra dsRNA injected
Wild type
BP
WP
M
-ve (Bdgapdh)
3,000 bp
1,000 bp
500 bp
100 bp
400 bp
11
12

Supplementary Figure S9. Full-length gels showing the expression analysis of *Bdtra* dsRNA treated and wild-type flies. RT-PCRs of (a) *Bdtra* and *Bddsx*^{M}, (b) *Bddsx*^{F} and *Bdfru*^{M}, (c) *Bdtra-2*, and (d) *Bdgapdh* were carried out to analyse the switch of the splicing pattern from female to male of *Bdtra* dsRNA injected pseudomales. The expression patterns of wild-type brown-pupae (BP) males and white-pupae (WP) females were compared with the *Bdtra* dsRNA treated brown-pupae males and white-pupae pseudomales. Lane -ve was used as a negative control for each experiment. M is the 100 bp DNA ladder plus, ranging from 100 to 3,000 bp. The primers used in this experiment are shown in Supplementary Tables S5 and S6. The red boxes represent regions in the original gels that were cropped and represented in Figure 4. The numbers on the top left hand corner of the red boxes denote different cropped regions. However, the numerical order was not necessarily arranged according to the cropped layout in the main figure. Similar gel labels were used to assist localisation of the red boxes in Figure 4.

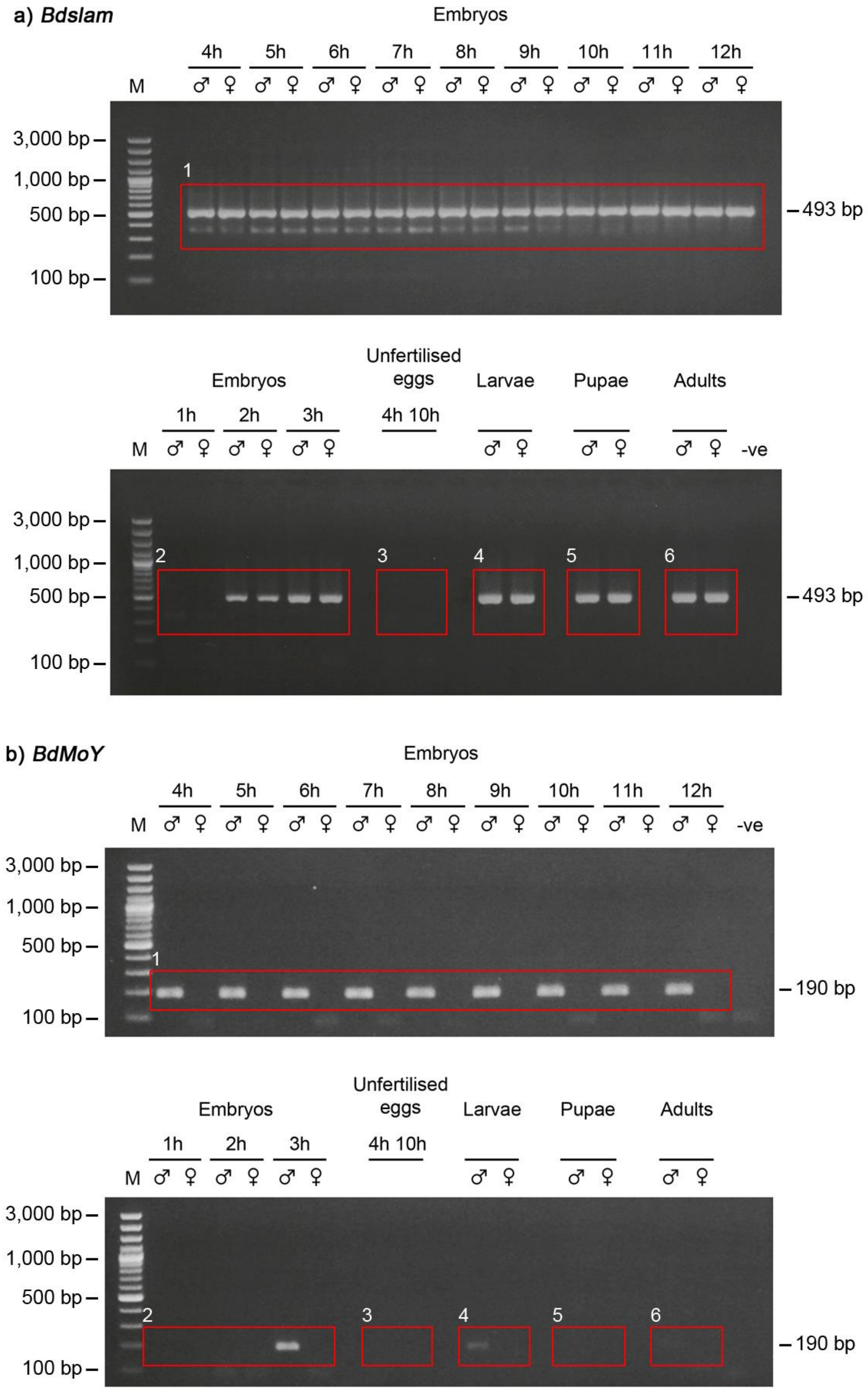
a) Bdslam
Embryos
4h 5h 6h 7h 8h 9h 10h 11h 12h
M ♂ ♀ ♂ ♀ ♂ ♀ ♂ ♀ ♂ ♀ ♂ ♀ ♂ ♀ ♂ ♀ ♂ ♀
3,000 bp
1,000 bp
500 bp
100 bp
1
493 bp
Embryos
Unfertilised eggs
Larvae
Pupae
Adults
1h 2h 3h
4h 10h
M ♂ ♀ ♂ ♀ ♂ ♀ ♂ ♀ ♂ ♀ ♂ ♀ -ve
3,000 bp
1,000 bp
500 bp
100 bp
2 3 4 5 6
493 bp
b) BdMoY
Embryos
4h 5h 6h 7h 8h 9h 10h 11h 12h
M ♂ ♀ ♂ ♀ ♂ ♀ ♂ ♀ ♂ ♀ ♂ ♀ ♂ ♀ ♂ ♀ ♂ ♀ -ve
3,000 bp
1,000 bp
500 bp
100 bp
1
190 bp
Embryos
Unfertilised eggs
Larvae
Pupae
Adults
1h 2h 3h
4h 10h
M ♂ ♀ ♂ ♀ ♂ ♀ ♂ ♀ ♂ ♀ ♂ ♀
3,000 bp
1,000 bp
500 bp
100 bp
2 3 4 5 6
190 bp

c) ***Bdtra***

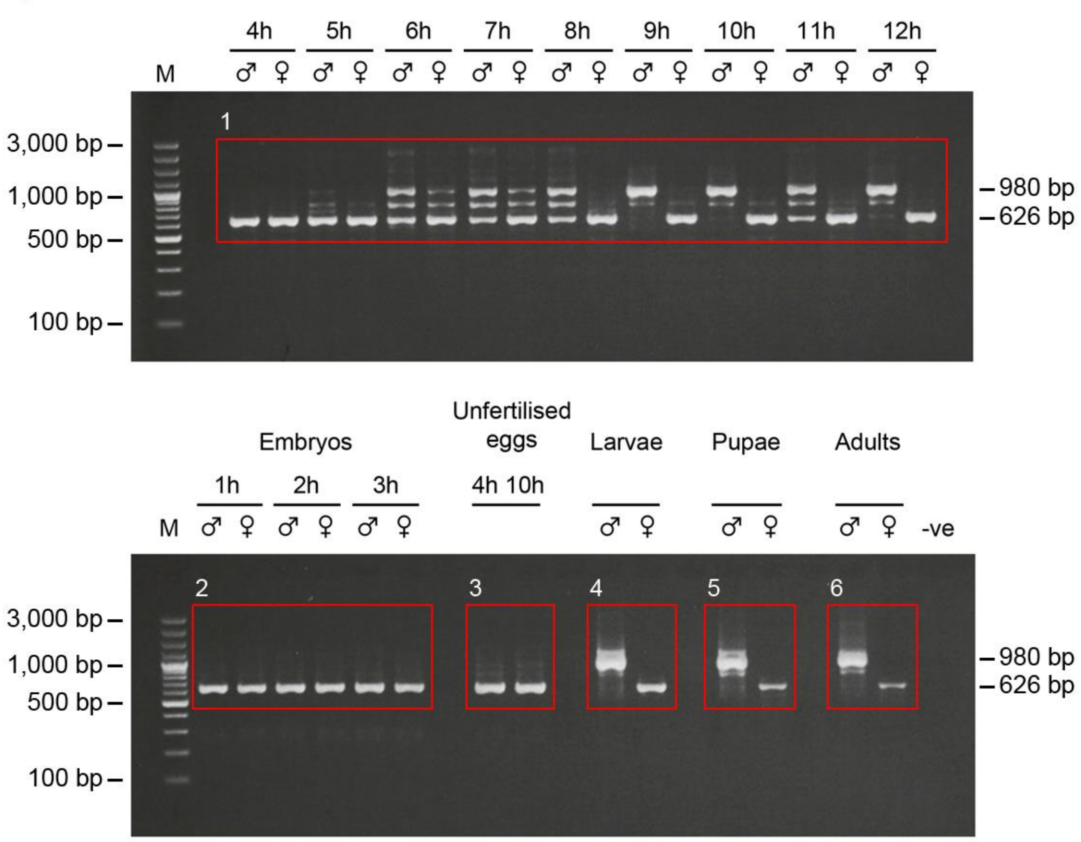

d) ***Bdtra-2***

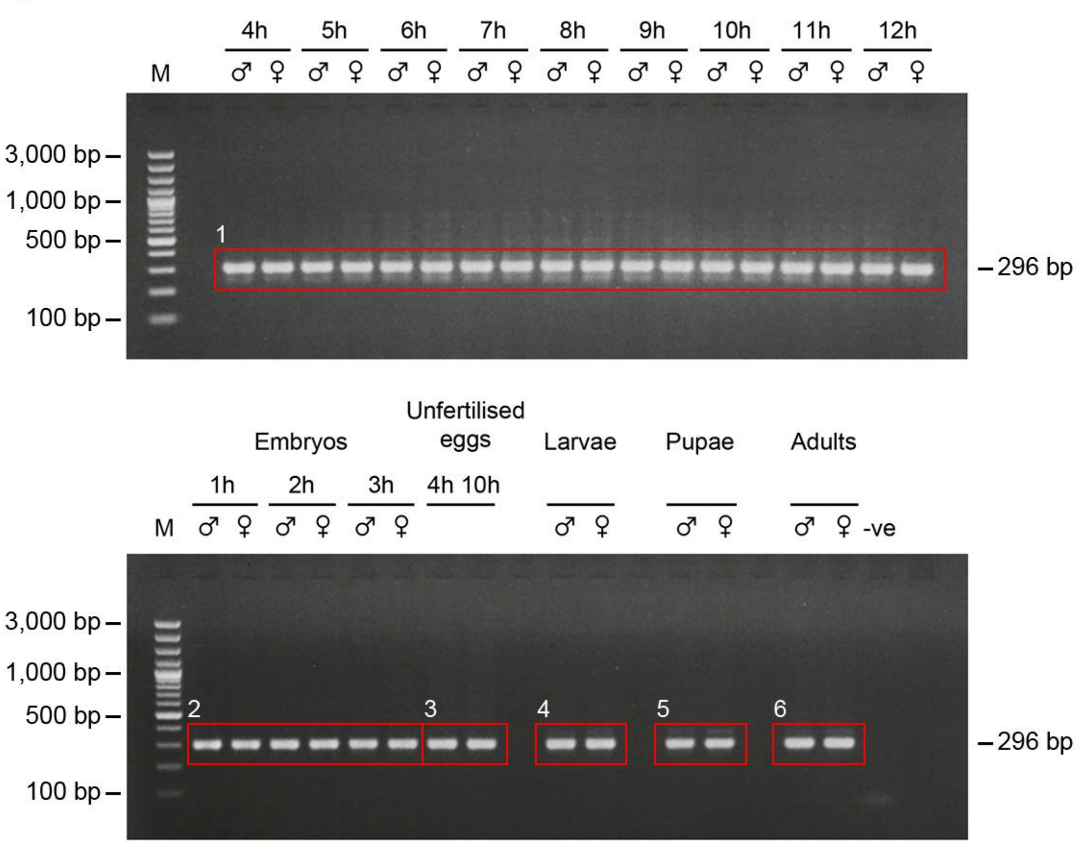

e) ***Bddsx***^{F}

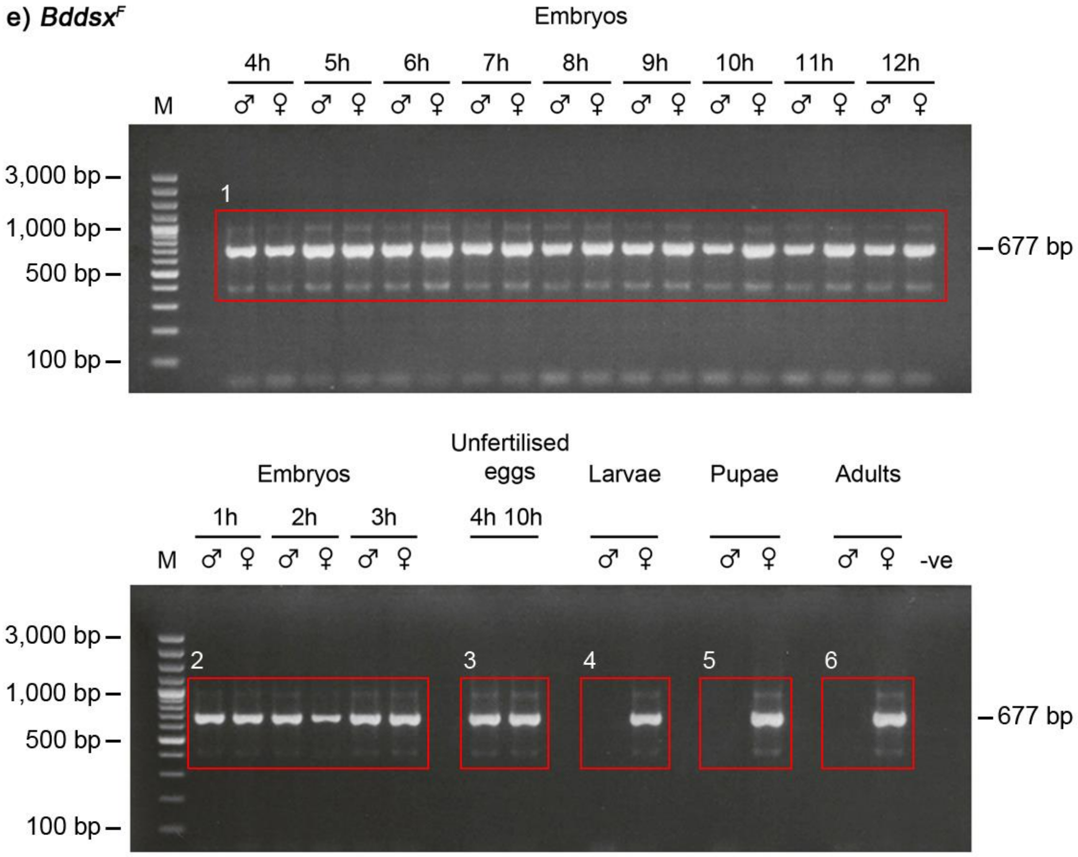

f) ***Bddsx***^{M}

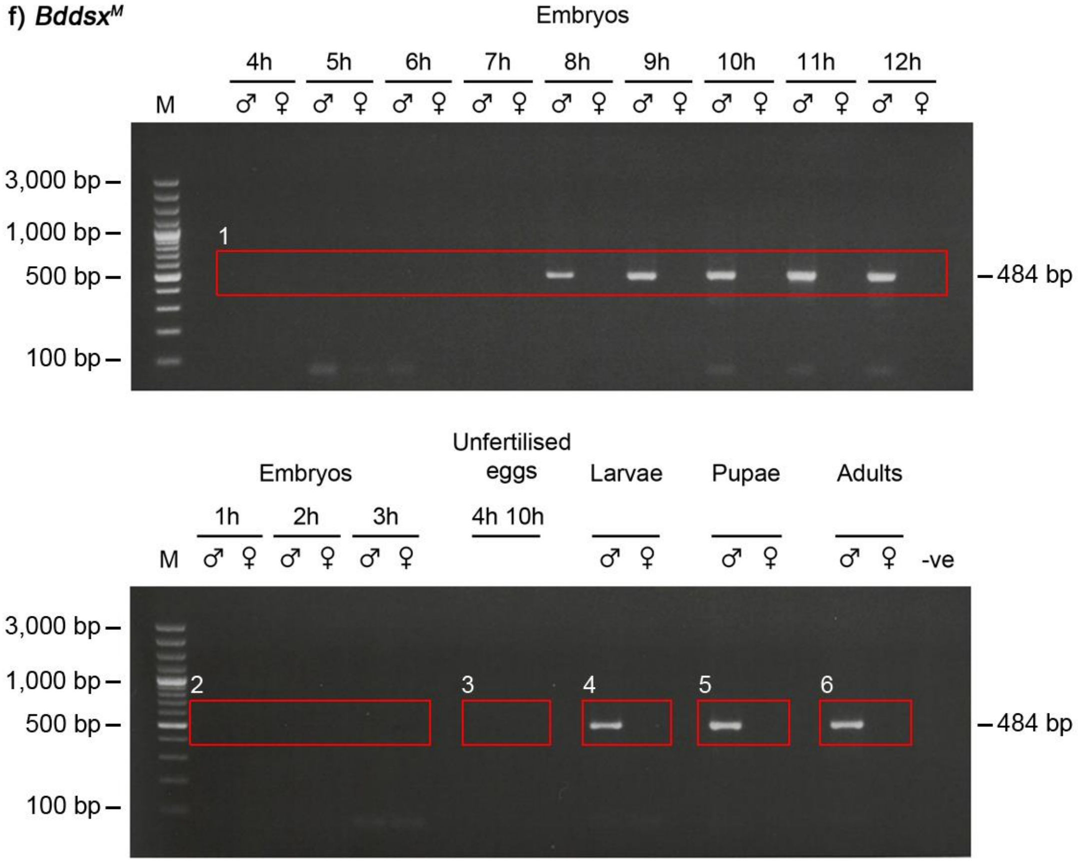

g) ***Bdfru***^{M}

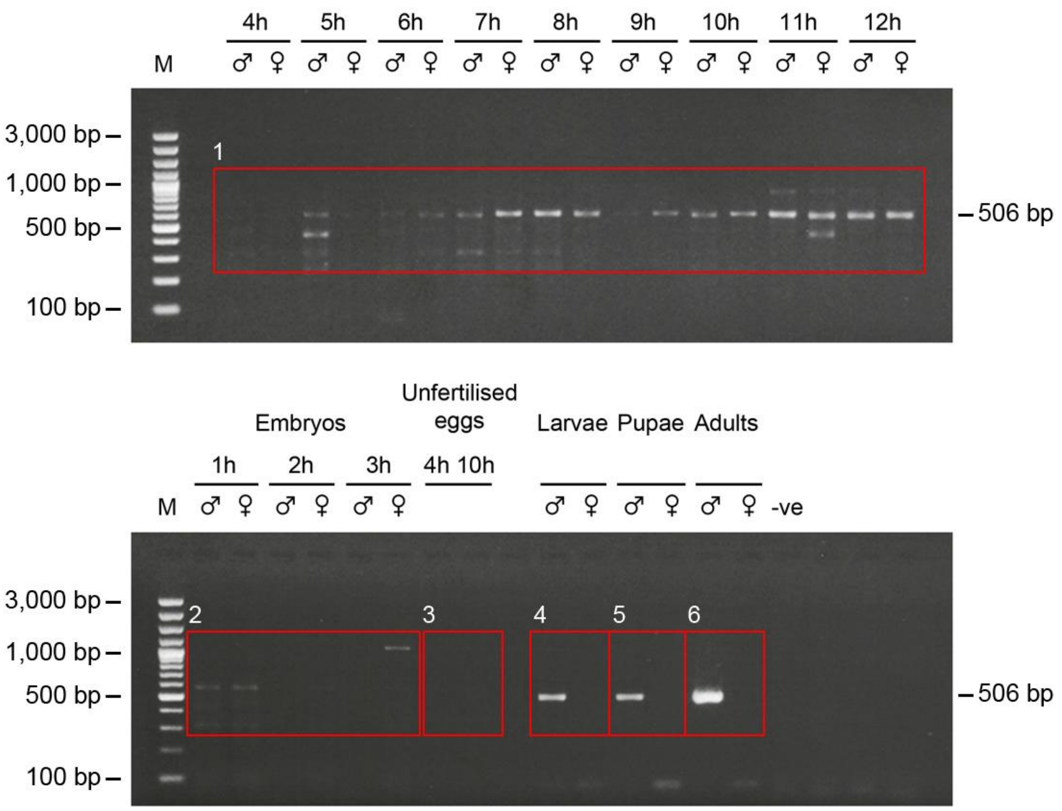

h) ***Bdgapdh***

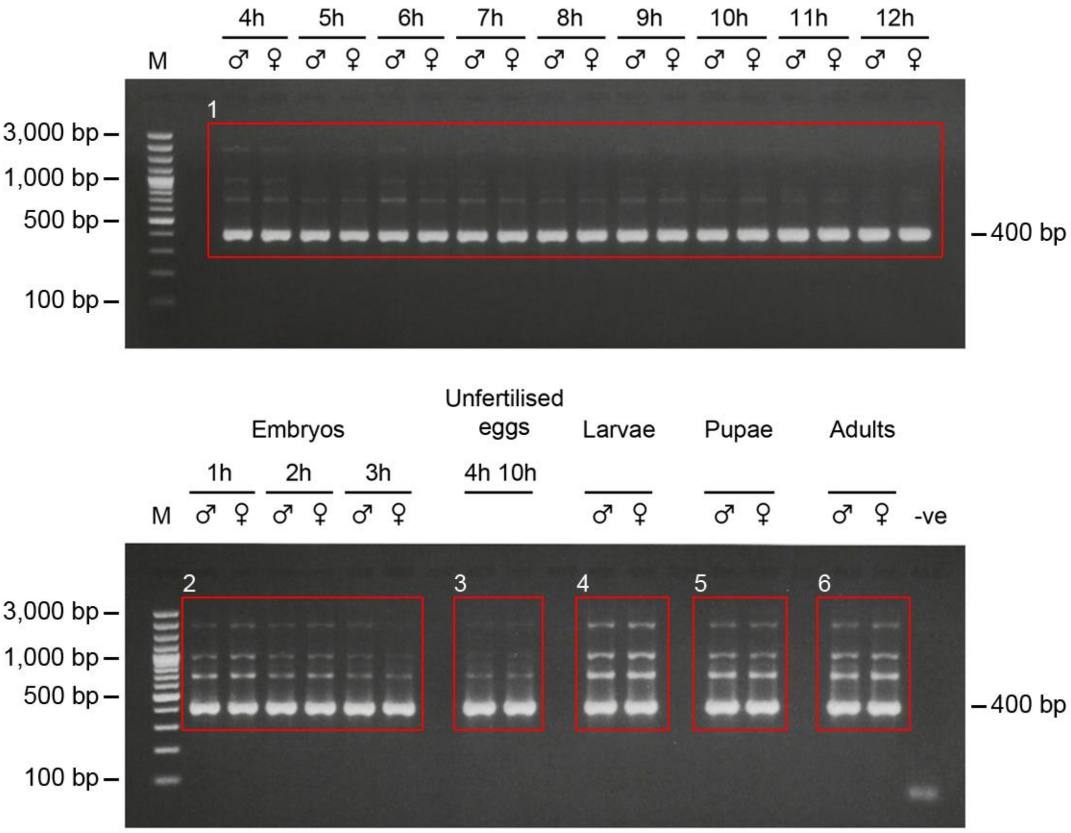

i) ***Bcslam***

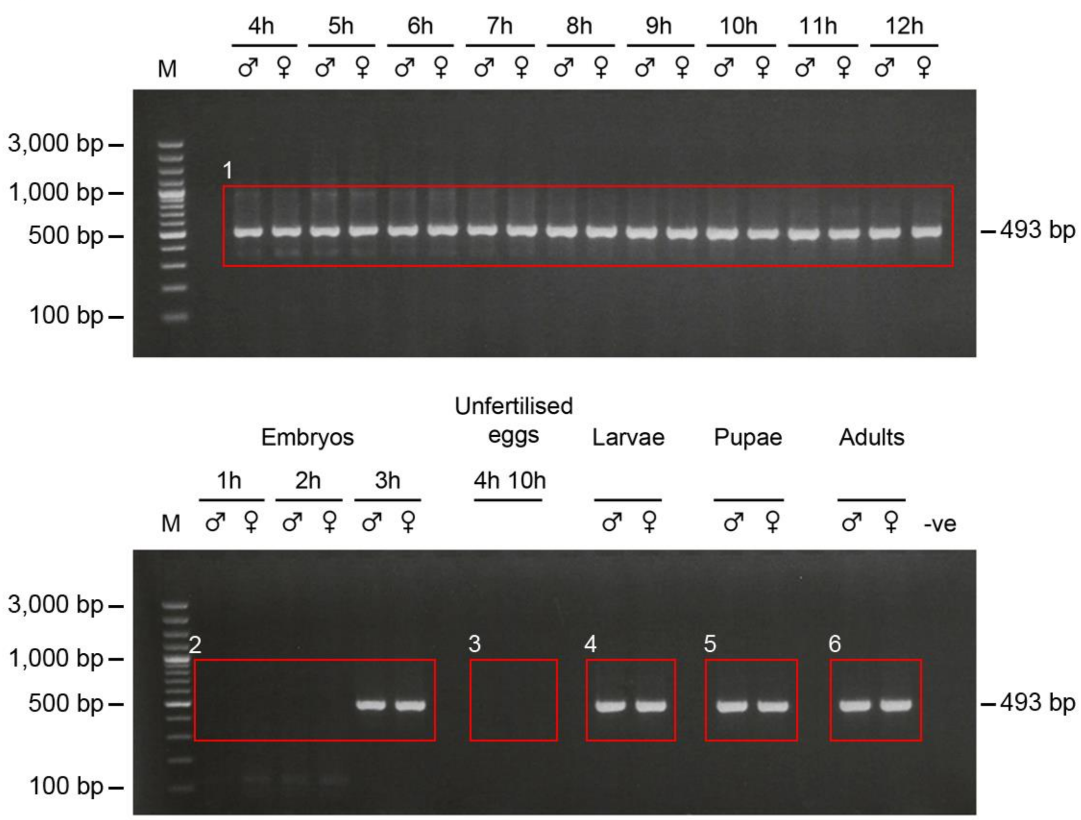

j) ***BcMoY***

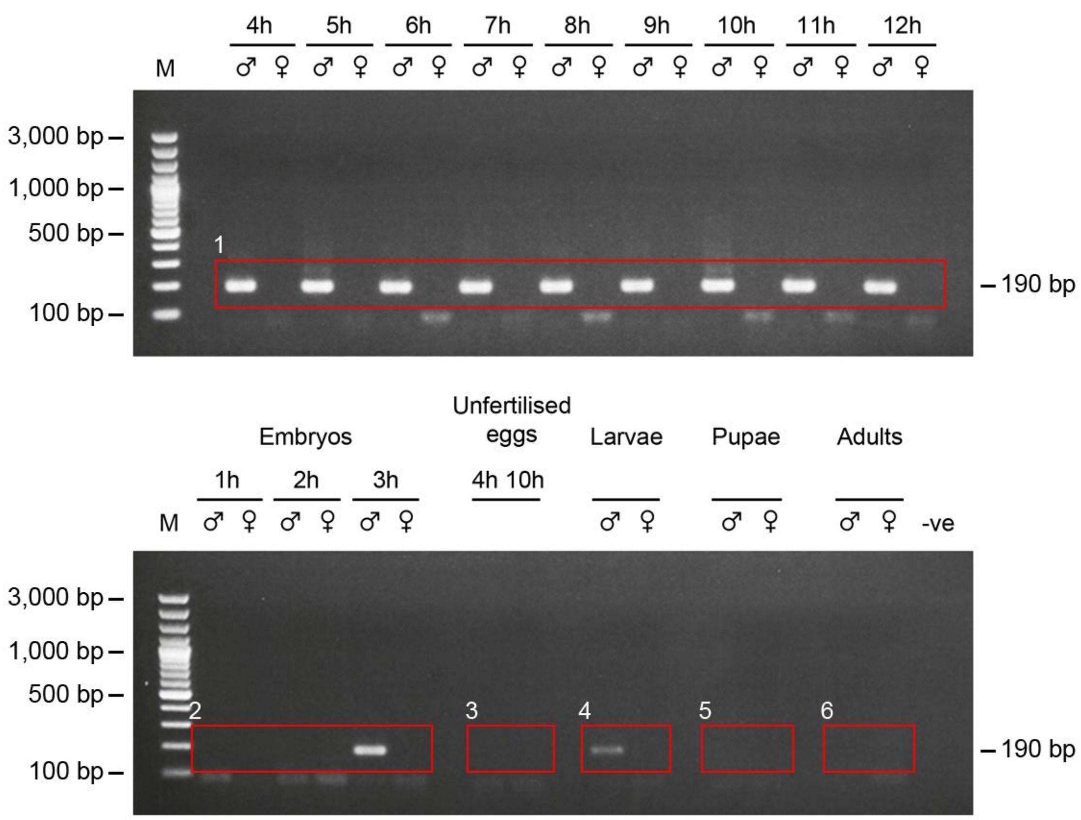

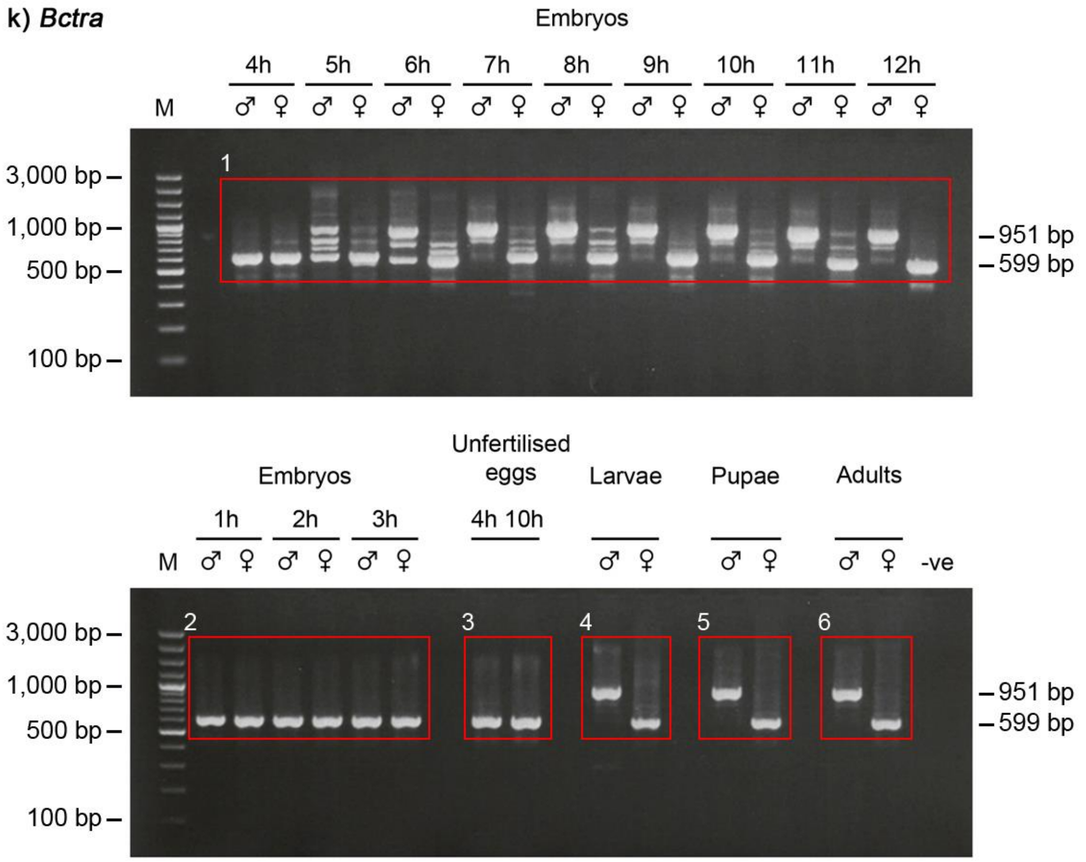
k) Bctra
Embryos
4h 5h 6h 7h 8h 9h 10h 11h 12h
M ♂ ♀ ♂ ♀ ♂ ♀ ♂ ♀ ♂ ♀ ♂ ♀ ♂ ♀ ♂ ♀ ♂ ♀
3,000 bp
1,000 bp
500 bp
100 bp
1
951 bp
599 bp
Embryos
Unfertilised eggs
Larvae
Pupae
Adults
1h 2h 3h
4h 10h
M ♂ ♀ ♂ ♀ ♂ ♀ ♂ ♀ ♂ ♀ ♂ ♀ -ve
3,000 bp
1,000 bp
500 bp
100 bp
2
3
4
5
6
951 bp
599 bp

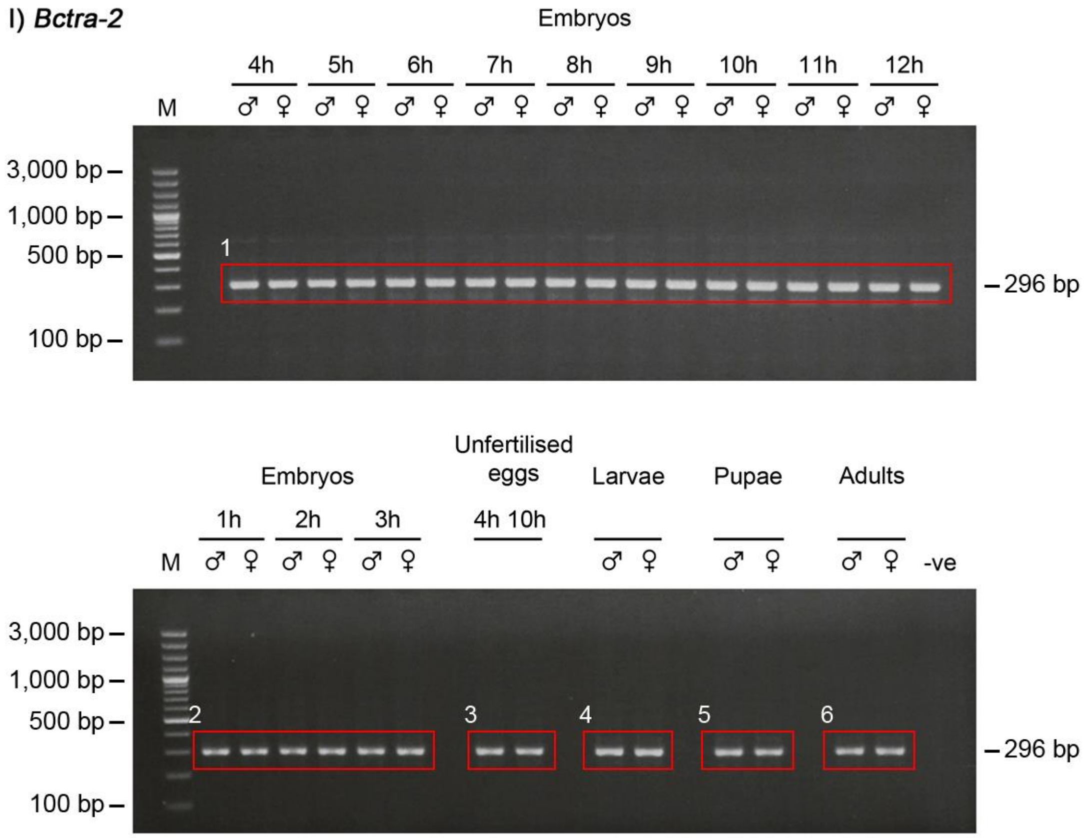
l) Bctra-2
Embryos
4h 5h 6h 7h 8h 9h 10h 11h 12h
M ♂ ♀ ♂ ♀ ♂ ♀ ♂ ♀ ♂ ♀ ♂ ♀ ♂ ♀ ♂ ♀ ♂ ♀
3,000 bp
1,000 bp
500 bp
100 bp
1
296 bp
Embryos
Unfertilised eggs
Larvae
Pupae
Adults
1h 2h 3h
4h 10h
M ♂ ♀ ♂ ♀ ♂ ♀ ♂ ♀ ♂ ♀ ♂ ♀ -ve
3,000 bp
1,000 bp
500 bp
100 bp
2
3
4
5
6
296 bp

m) ***Bcdsx***^{F}

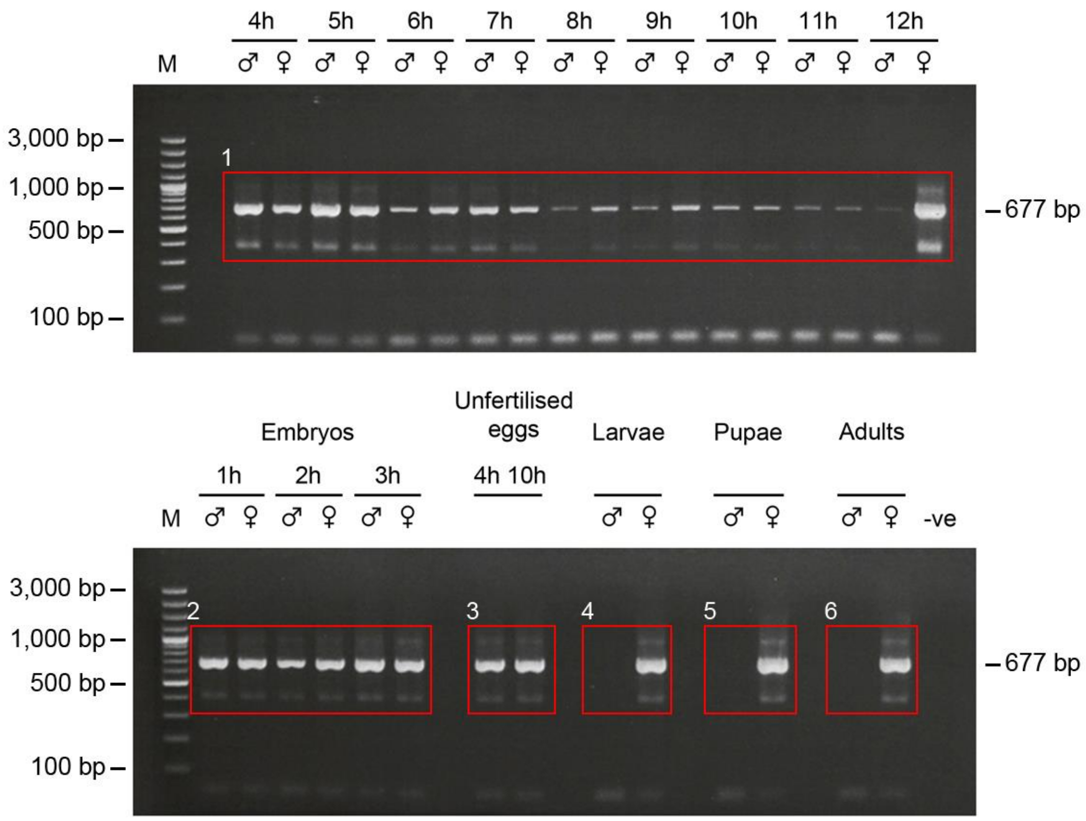

n) ***Bcdsx***^{M}

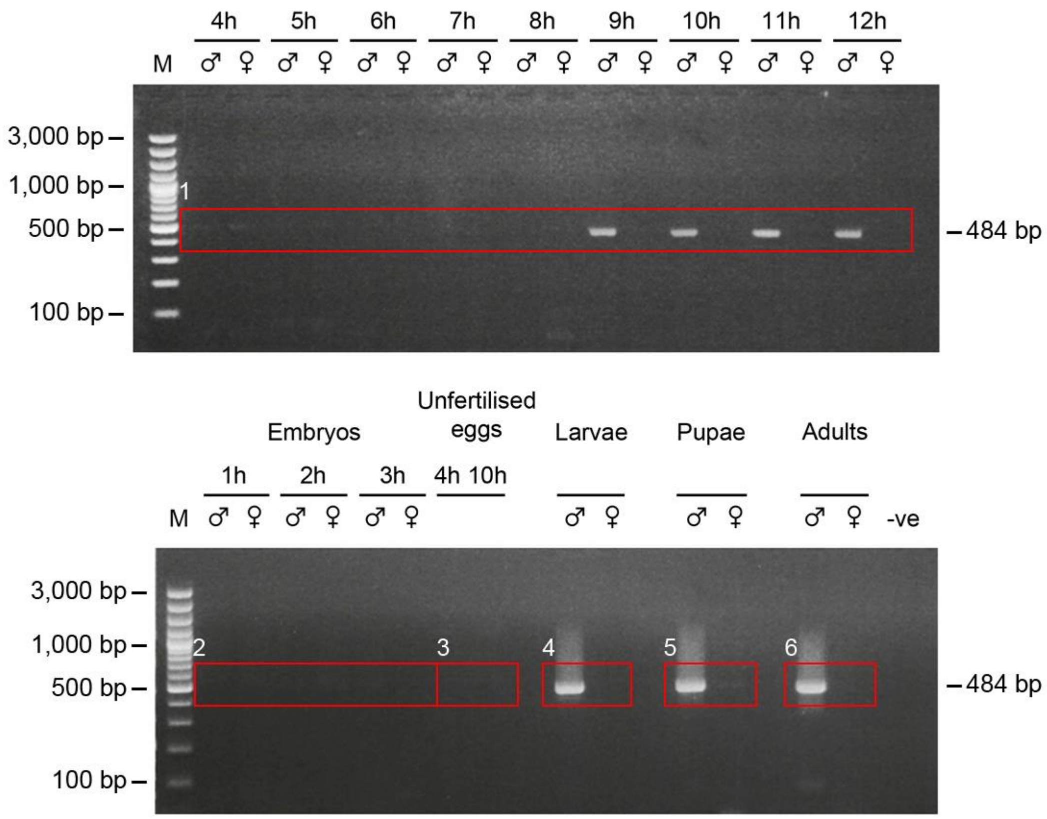

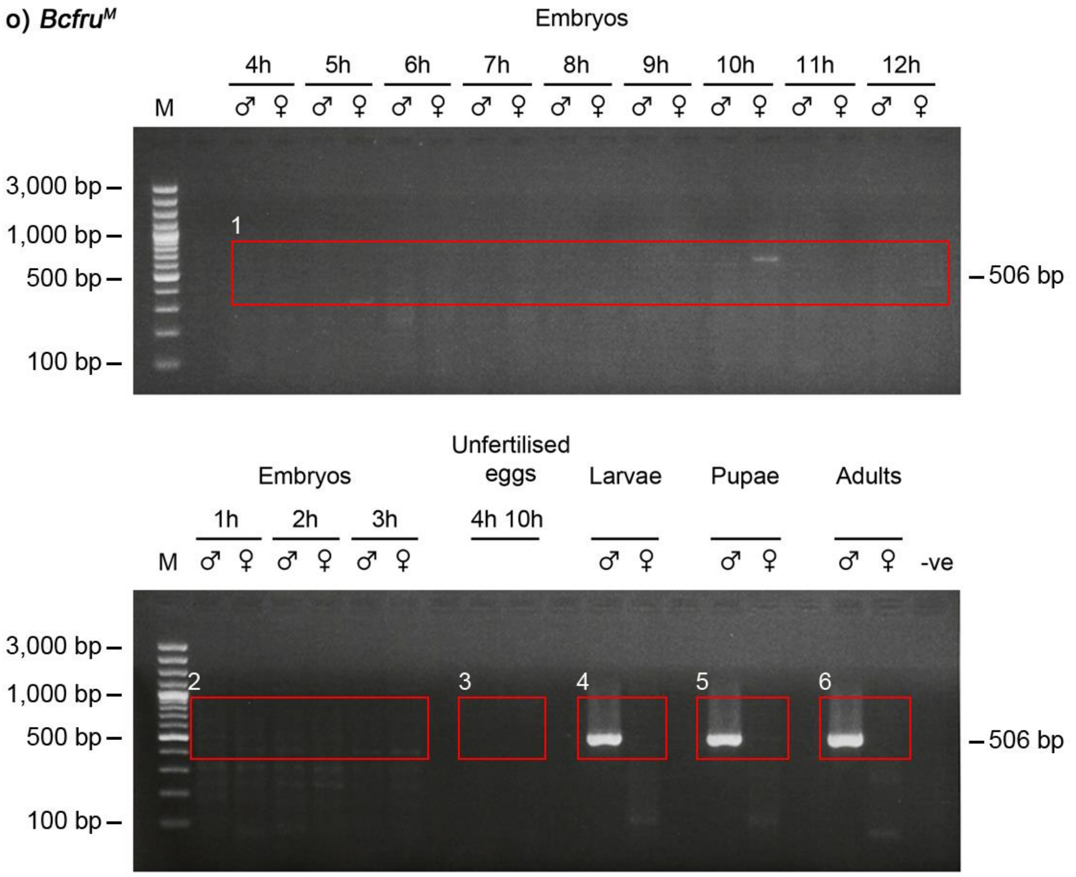
o) BcfruM
Embryos
4h 5h 6h 7h 8h 9h 10h 11h 12h
M ♂ ♀ ♂ ♀ ♂ ♀ ♂ ♀ ♂ ♀ ♂ ♀ ♂ ♀ ♂ ♀ ♂ ♀
3,000 bp
1,000 bp
500 bp
100 bp
1
506 bp
Embryos
Unfertilised eggs
Larvae
Pupae
Adults
1h 2h 3h
4h 10h
M ♂ ♀ ♂ ♀ ♂ ♀ ♂ ♀ ♂ ♀ ♂ ♀ -ve
3,000 bp
1,000 bp
500 bp
100 bp
2
3
4
5
6
506 bp

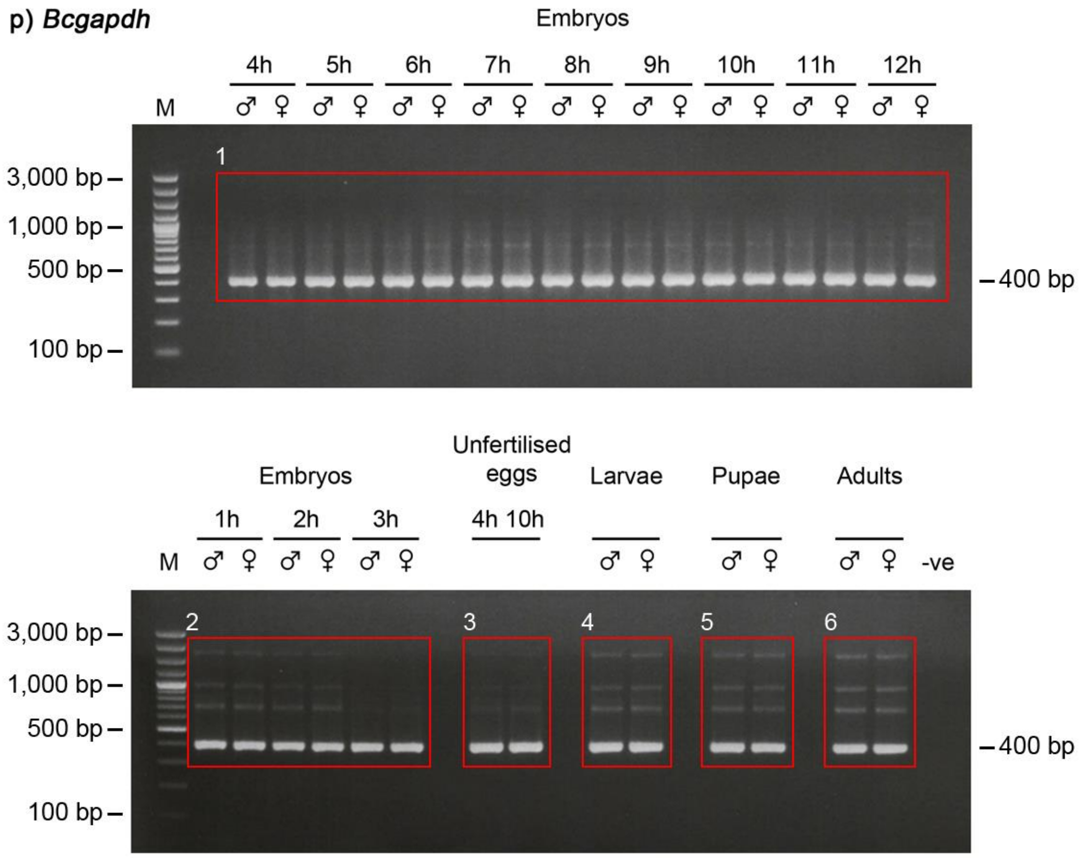
p) Bcgapdh
Embryos
4h 5h 6h 7h 8h 9h 10h 11h 12h
M ♂ ♀ ♂ ♀ ♂ ♀ ♂ ♀ ♂ ♀ ♂ ♀ ♂ ♀ ♂ ♀ ♂ ♀
3,000 bp
1,000 bp
500 bp
100 bp
1
400 bp
Embryos
Unfertilised eggs
Larvae
Pupae
Adults
1h 2h 3h
4h 10h
M ♂ ♀ ♂ ♀ ♂ ♀ ♂ ♀ ♂ ♀ ♂ ♀ -ve
3,000 bp
1,000 bp
500 bp
100 bp
2
3
4
5
6
400 bp

Supplementary Figure S10. Full-length gels showing the expression analysis of sex-determining genes during the early stages of embryogenesis and other developmental stages in *B. dorsalis* (a-h) and *B. correcta* (i-p). The samples were prepared from unfertilised eggs, embryos (1 to 12 h AEL), larvae, pupae, and adults. M is the 100 bp DNA ladder plus, ranging from 100 to 3,000 bp. Lane -ve was used as a negative control for each experiment. The primers used in this experiment are in Supplementary Tables S5 and S6. The red boxes represent regions in the original gels that were cropped and represented in Figure 5. The numbers on the top left hand corner of the red boxes denote different cropped regions. However, the numerical order was not necessarily arranged according to the cropped layout in the main figure. Similar gel labels were used to assist localisation of the red boxes in Figure 5.

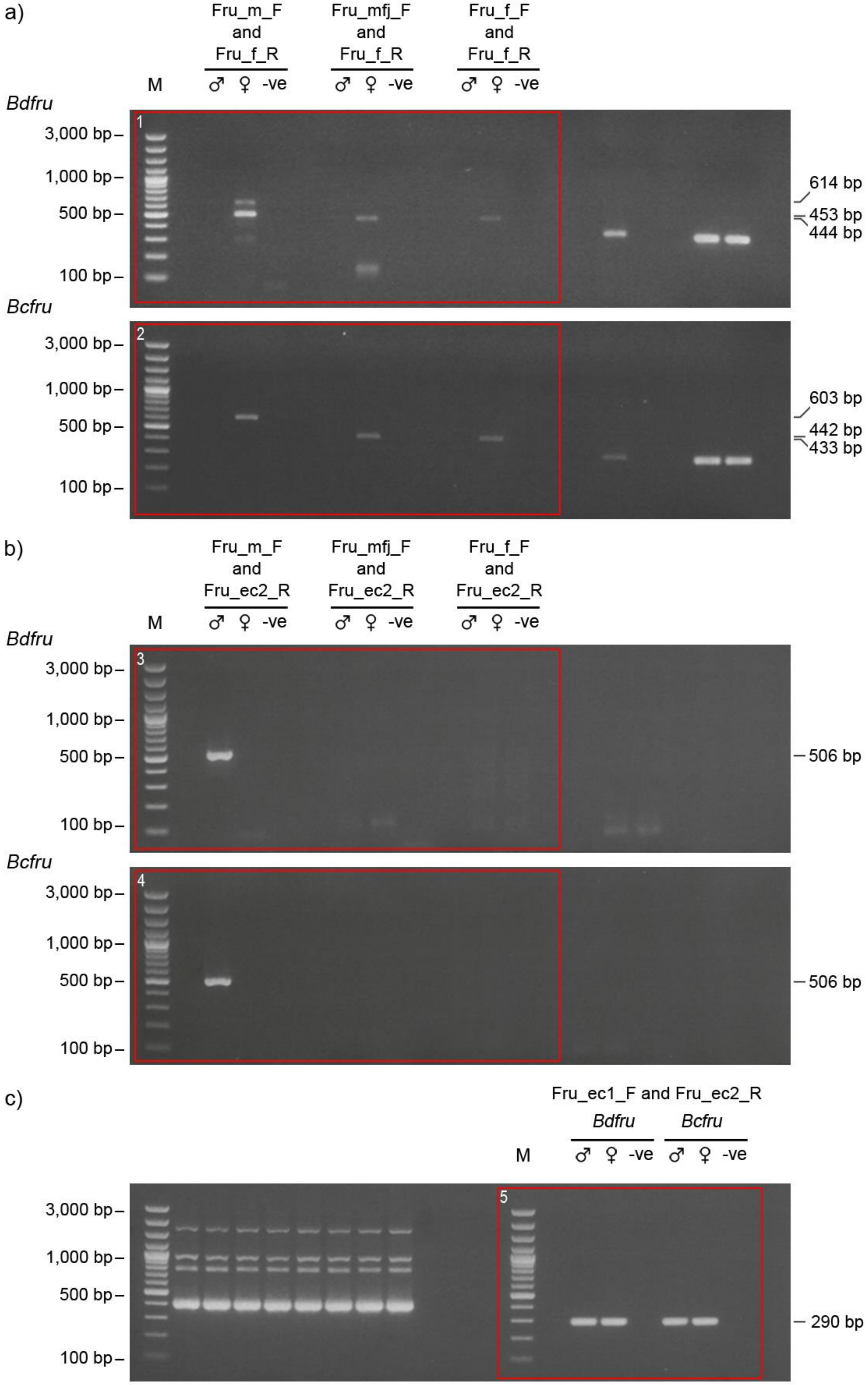
a)
Fru_m_F and Fru_f_R
Fru_mfj_F and Fru_f_R
Fru_f_F and Fru_f_R
M
♂ ♀ -ve
Bdfru
Bcfru
3,000 bp
1,000 bp
500 bp
100 bp
614 bp
453 bp
444 bp
603 bp
442 bp
433 bp
b)
Fru_m_F and Fru_ec2_R
Fru_mfj_F and Fru_ec2_R
Fru_f_F and Fru_ec2_R
506 bp
c)
Fru_ec1_F and Fru_ec2_R
290 bp
1
2
3
4
5

Supplementary Figure S11. Full-length gels showing the expression analysis of the putative F exon and the M exon are shown here. Two RT-PCR experiments were carried out to identify (a) the presence of the putative F exon and the connection to the M exon and (b) the connection between the putative F exon and the BTB encoding exons. The common C1 and C2 exons was used as a positive control (c). The cDNA samples were prepared from male and female adult heads. M is the 100 bp DNA ladder plus, ranging from 100 to 3,000 bp. Lane -ve was used as a negative control for each experiment. The primers used in this experiment are shown in Supplementary Table S4. The red boxes represent regions in the original gels that were cropped and represented in Supplementary Figure S5. The numbers on the top left hand corner of red boxes denote different cropped regions. However, the numerical order was not necessarily arranged according to the cropped layout. Similar gel labels were used to assist localisation of the red boxes in Supplementary Figure S5.

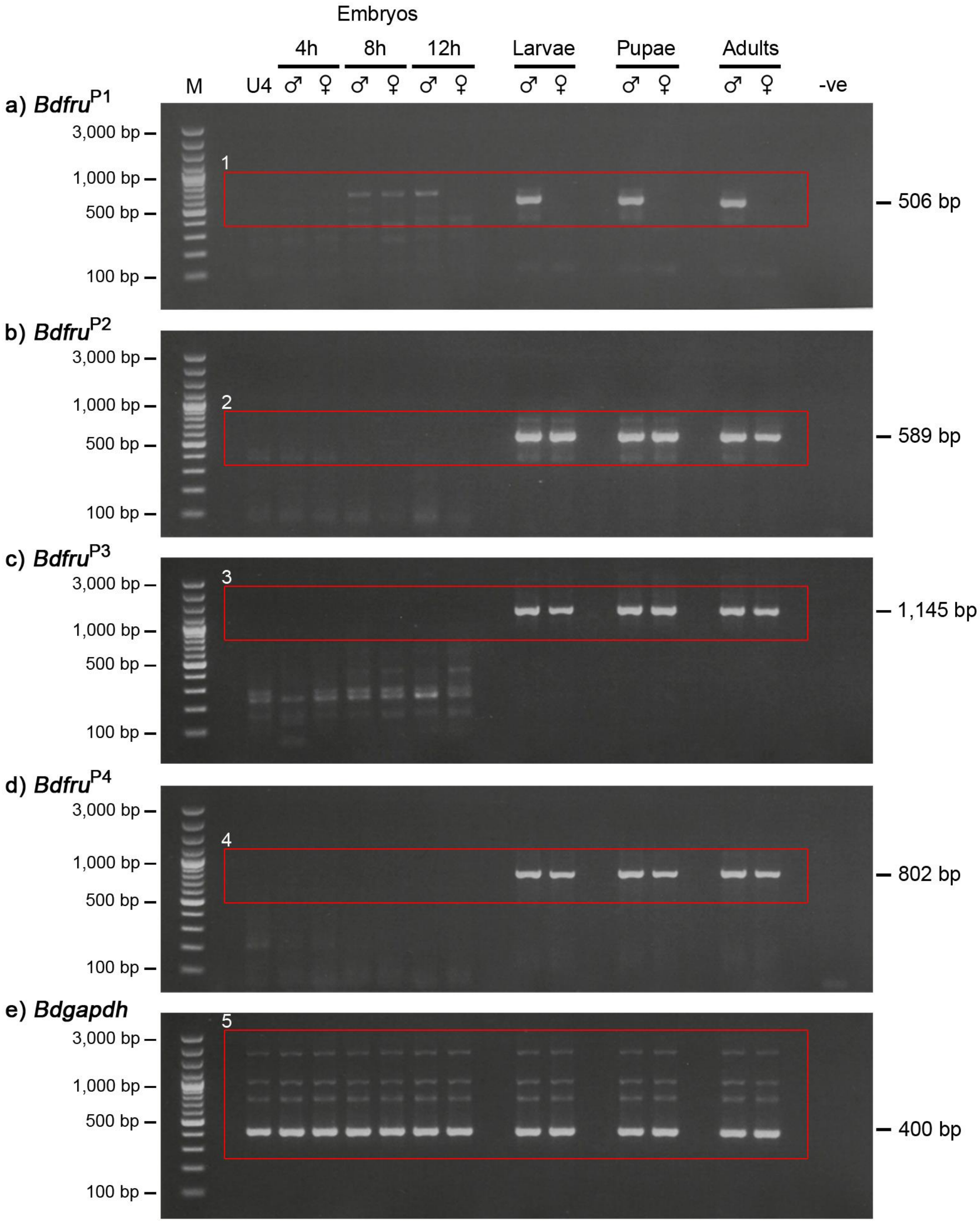
Embryos
4h
8h
12h
Larvae
Pupae
Adults
M
U4
♂
♀
-ve
a) BdfruP1
3,000 bp
1,000 bp
500 bp
100 bp
1
506 bp
b) BdfruP2
2
589 bp
c) BdfruP3
3
1,145 bp
d) BdfruP4
4
802 bp
e) Bdgapdh
5
400 bp

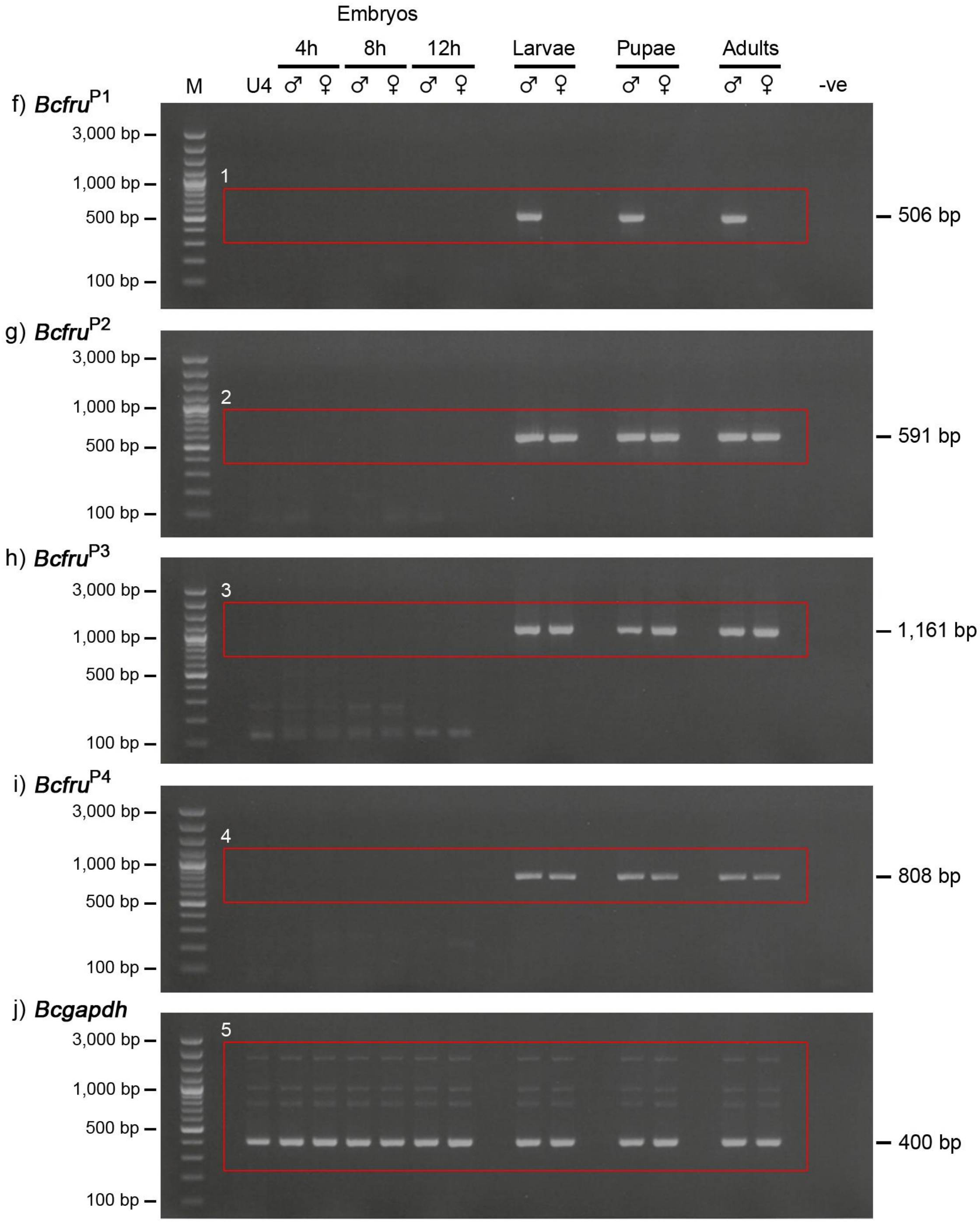

Supplementary Figure S12. Full-length gels showing the expression analysis of the P1 to P4 transcripts during developmental stages of *Bdfru* (a-e) and *Bcfru* (f-j) genes. The samples were prepared from unfertilised eggs (4 h AEL), embryos (4, 8, and 12 h AEL), larvae, pupae, and adults. M is the 100 bp DNA ladder plus, ranging from 100 to 3,000 bp. Lane -ve was used as a negative control for each experiment. The primers used in this experiment are in Supplementary Table S4. The red boxes represent regions in the original gels that were cropped and represented in Supplementary Figure S8. The numbers on the top left hand corner of red boxes denote different

cropped regions. The numerical order was arranged according to the cropped layout in Supplementary Figure S8.

References

1. Ito, H. *et al.* Sexual orientation in *Drosophila* is altered by the satori mutation in the sex-determination gene *fruitless* that encodes a zinc finger protein with a BTB domain. *Proc Natl Acad Sci USA* **93**, 9687-9692 (1996).
2. Ryner, L. C. *et al.* Control of Male Sexual Behavior and Sexual Orientation in *Drosophila* by the *fruitless* Gene. *Cell* **87**, 1079-1089 (1996).
3. Persikov, A. V., Rowland, E. F., Oakes, B. L., Singh, M. & Noyes, M. B. Deep sequencing of large library selections allows computational discovery of diverse sets of zinc fingers that bind common targets. *Nucleic Acids Res* **42**, 1497-1508 (2014).
4. Persikov, A. V. *et al.* A systematic survey of the Cys_2His_2 zinc finger DNA-binding landscape. *Nucleic Acids Res* **43**, 1965-1984 (2015).
5. Heinrichs, V., Ryner, L. C. & Baker, B. S. Regulation of sex-specific selection of *fruitless* 5' splice sites by *transformer* and *transformer-2*. *Mol Cell Biol* **18**, 450-458 (1998).
6. Gailey, D. A. *et al.* Functional Conservation of the *fruitless* Male Sex-Determination Gene Across 250 Myr of Insect Evolution. *Mol Biol Evol* **23**, 633-643 (2005).
7. Meier, N. *et al.* Genetic control of courtship behavior in the housefly: evidence for a conserved bifurcation of the sex-determining pathway. *PLoS One* **8**, e62476-e62476, doi:10.1371/journal.pone.0062476 (2013).
