# Supplementary material for: The transformer-2 and fruitless characterisation with developmental expression profiles of sex-determining genes in Bactrocera dorsalis and B. correcta
Source: Sci Rep. 2020 Oct 21;10:17938. doi: 10.1038/s41598-020-74856-6 (PMC7578103; doi:10.1038/s41598-020-74856-6)
Supplement: Supplementary file 2 — Supplementary Tables. [file 41598_2020_74856_MOESM2_ESM.pdf]

Title: The *transformer-2* and *fruitless* characterisation with developmental expression profiles of sex-determining genes in *Bactrocera dorsalis* and *B. correcta*

Kamoltip Laohakieat[1], Siriwan Isasawin[1], Sujinda Thanaphum[1,*]

[1] Regional R&D Training Center for Insect Biotechnology (RCIB), Department of Biotechnology, Faculty of Science, Mahidol University, Rama VI Road, Bangkok 10400, Thailand

[*]corresponding author: sujinda.tha@mahidol.ac.th

Supplementary Table S1. Identity percentages from the pairwise comparison of Transformer-2 (TRA-2) amino acid sequences

Family			Tephritidae										Drosophilidae
	Genus		*Bactrocera*					*Zeugodacus*	*Anastrepha*			*Ceratitis*	*Drosophila*
		Species	*Bd*	*Bc*	*Bj*	*Bt*	*Bo*	*Zcu*	*Ao*	*Af*	*As*	*Cc*	*Dm*
Tephritidae	*Bactrocera*	*Bd*											
		Bc	98										
		Bj	98	98									
		Bt	97	97	98								
		Bo	96	96	96	95							
	Zeugodacus	*Zcu*	96	95	96	94	94						
	Anastrepha	*Ao*	89	88	88	87	86	89					
		Af	89	89	88	87	87	89	98				
		As	89	88	88	87	86	89	97	98			
	Ceratitis	*Cc*	91	91	90	89	89	91	89	89	89		
Drosophilidae	*Drosophila*	*Dm*	45	44	45	43	45	43	42	42	42	44	
Number of amino acids			251	251	251	255	251	250	249	249	249	251	264

The pairwise comparisons were performed within the same genus, *Bactrocera* or *Anastrepha*, among different genera of the family Tephritidae, and with *D. melanogaster* from the family *Drosophilidae*. Fruit fly species are indicated as the following: *Bd, B. dorsalis*; *Bc, B. correcta*; *Bj, B. jarvisi*; *Bt, B. tryoni*; *Bo, B. oleae*; *Zcu, Z. cucurbitae*; *Ao, A. obliqua*; *Af, A. fraterculus aff.1*; *As, A. suspensa*; *Cc, C. capitata*; *Dm, D. melanogaster*. GenBank Acc. Nos. of TRA-2 amino acid sequences are presented in Supplementary Table S3.

Supplementary Table S2. The connection between transcript classes and zinc-finger exons of *Bdfru* and *Bcfru*

fru transcript classes	Zinc-finger exons							
	Male				Female			
	A	B	C	D	A	B	C	D
P1 (exon M)	✓	✓	✓	-	-	-	-	-
P2 (exon U1)	✓	✓	✓	✓	✓	✓	✓	✓
P3 (exons U2 and U4)	✓	✓	✓	✓	✓	✓	✓	✓
P4 (exon U3)	✓	-	✓	✓	✓	-	✓	✓

Supplementary Table S3. GenBank accession numbers of *transformer-2* (*tra-2*) and *fruitless* (*fru*) sequences used in this study

Species	GenBank accession numbers	References
transformer-2		
Bactrocera dorsalis	MT900512, MT900514	In this study
Bactrocera correcta	MT900513, MT900515	In this study
Bactrocera dorsalis	KP342061	1
Bactrocera oleae	AJ547623	-
Bactrocera tryoni	KJ443723	2
Bactrocera jarvisi	KJ443722	2
Zeugodacus cucurbitae	KR056217	3
Anastrepha obliqua	FN658607	4
Anastrepha fraterculus aff.1	FN658608	4
Anastrepha suspensa	JN597290	5
Ceratitis capitata	EU999754	6
Drosophila melanogaster	NM_057416	-
fruitless		
Bactrocera dorsalis	MT900516 - MT900519, MT900524 - MT900528	In this study
Bactrocera correcta	MT900520 - MT900523, MT900529 - MT900533	In this study
Bactrocera dorsalis	XM_011202587 - XM_011202592	-
Bactrocera oleae	XM_014238789 - XM_014238790	-
Bactrocera latifrons	XM_018927950 - XM_018927953	-
Zeugodacus cucurbitae	XM_011191466 - XM_011191472	-
Ceratitis capitata	EU999755	6
	XM_020861837 - XM_020861839	-
Musca domestica	KC750909 - KC750915	7
Drosophila melanogaster	NM_169820, NM_001275783, NM_206514, NM_169816, NM_169822	-
Anopheles gambiae	AY785361, AY785360, AY725820, AY725819	8
Tribolium castaneum	JQ409470, JQ409471, JQ409472, XM_008202773	-
Nasonia vitripennis	GQ338170, GQ338167, NM_001164125, NM_001164122, NM_001164128, NM_001164123, NM_001164130, NM_001164134, GQ338183.1	9

Supplementary Table S4. Primers used in this study for isolation of *transformer-2* (*tra-2*) and *fruitless* (*fru*) genes

Purpose	Primer name	Sequence (5' → 3')
Isolation of *tra-2* transcripts		
RT-PCR	Tra-2_e2F	CGTAGCCGCAGTATTTCCC
	Tre-2_e6aR	AGTGTGCGGTCTTTGCGTTG
5'RACE	UPM_long	CTAATACGACTCACTATAGGGCAAGCAGTGGTATCAACGCAGAGT
	UPM_short	CTAATACGACTCACTATAGGGC
	Tra-2_e6bR	TTGCGACTGTGATAAGGTGAG
3'RACE	Tra-2_e2F	CGTAGCCGCAGTATTTCCC
	UPM_long	CTAATACGACTCACTATAGGGCAAGCAGTGGTATCAACGCAGAGT
	UPM_short	CTAATACGACTCACTATAGGGC
Isolation of *tra-2* exon/intron sequences		
PCR	Tra-2_F	AGCATATCGAGCGAGTCCAT
	Tra-2_e2R	AACTGGCGACTTGGTGTAAC
	Tra-2_e2F	CGTAGCCGCAGTATTTCCC
	Tre-2_e6aR	AGTGTGCGGTCTTTGCGTTG
	Tra-2_e5F	GGACCCATTGAACGAATACAAG
	Tra-2_e7R	CAGAGTAAATGCCAAACATGG
Isolation of *fru* transcripts		
RT-PCR	Fru_ec1F1	TGGAACAATCACCCGACGAA
	Fru_ec4R2	GCATGACTGAGACCAATACTA
	Fru_ec4F2	TAGTATTGGTCTCAGTCATGC
	Fru_zAR-2	ACATAATGACTGAAGAAACGATC
	Fru_zBR	TATCATTCACTTGTCGCATTGT
	Fru_zCR	GAGTGAAAGTGATGCCATCG
	Fru_zDR	TATCCTCGCCCACATTTATCG
Isolation of *fru* transcripts		
5'RACE	UPM_long	CTAATACGACTCACTATAGGGCAAGCAGTGGTATCAACGCAGAGT
	UPM_short	CTAATACGACTCACTATAGGGC

	Fru_ec3R2-AP	GATTACGCCAAGCTTGCTAACACTACGCTCACTATTCGCATCG
	Fru_ec3R1_nested-AP	GATTACGCCAAGCTTGCTCAAACTGCTGCTGCTGCGATGTG
	Fru_m_R	GGCACCTGTGCTCGGCTCGATGTCTGCA
3'RACE	UPM_long	CTAATACGACTCACTATAGGGCAAGCAGTGGTATCAACGCAGAGT
	UPM_short	CTAATACGACTCACTATAGGGC
	Fru_ec4F3L	GTAGCGGTAGTGTTAGTGCGATTCCCAGTAG
Study of the connection between transcript classes and zinc-finger encoding exons		
RT-PCR	Fru_m_F (P1)	ATGTTGGCCATGTCACAAGG
	Fru_5U-1 (P2)	GTGCTGAACGCCCAAAACTC
	Fru_5U-2 (P3)	TCAACTCGCATCGCATCATC
	Fru_5U-3 (P4)	TGGCGTATCGTAAAATGCTAAG
	Fru_ec2_R	GCTGTTTTGAGGAACATCGG
	Fru_zAF	GGACAACATTGATGGTGATGATGG
	Fru_zAR-2	ACATAATGACTGAAGAAACGATC
	Fru_zBF	TGTTATCTGCACCGCTGCAAACTG
	Fru_zBR	TATCATTCACTTGTCGCATTGT
	Fru_zCF	GCCCACCATCACATCTCTACAATC
	Fru_zCR	GAGTGAAAGTGATGCCATCG
	Fru_zDF	ATCGCATCGTTTGTGCTACGAACG
	Fru_zDR	TATCCTCGCCCACATTTATCG
Study of putative female-specific exon		
PCR and RT_PCR	Fru_m_F	ATGTTGGCCATGTCACAAGG
	Fru_mfj_F	GCCGCCGAGGTAAGGTTAC
	Fru_f_F	GTAAGGTTACGCCAAGCGGC
	Fru_f_R	AATGCTAGTAAAATGCGAGAGTGA
	Fru_ec1_F	TGGAACAATCACCCGACGAA
	Fru_ec2_R	GCTGTTTTGAGGAACATCGG
Study of the connection between transcript classes and developmental stages		
RT-PCR	Fru_m_F (P1)	ATGTTGGCCATGTCACAAGG
	Fru_5U-1 (P2)	GTGCTGAACGCCCAAAACTC

	Fru_5U-2 (P3)	TCAACTCGCATCGCATCATC
	Fru_5U-3 (P4)	TGGCGTATCGTAAAATGCTAAG
	Fru_ec2_R	GCTGTTTTGAGGAACATCGG

Supplementary Table S5. Schematic representation of relative locations and amplified regions of primers used for expression analysis of sex-determining genes in different developmental stages

Gene	Schematic representation of primer locations and amplified regions
slam	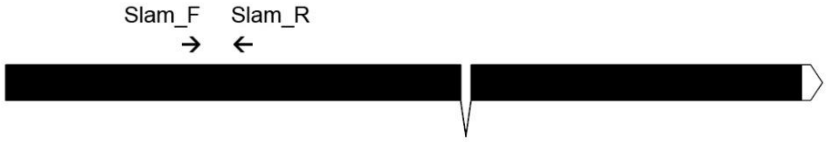
MoY	None
tra	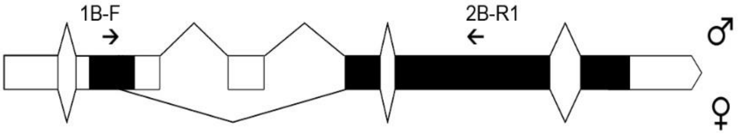
tra-2	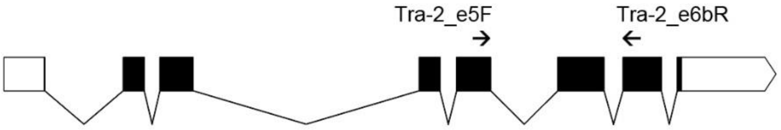
dsx	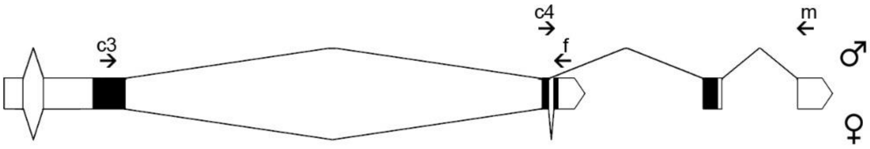
fru	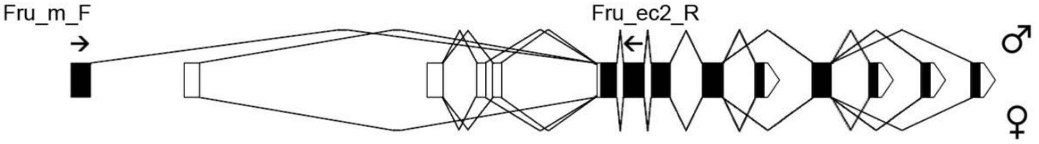
gapdh	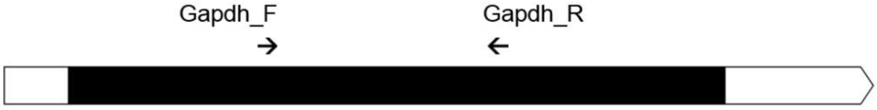

The representation of gene structure is not to scale. Boxes and lines represent exons and introns, respectively. The white and black regions indicate untranslated and translated regions, respectively. Arrows with names indicate primers used in the analysis. The sequences of these primers are in Supplementary Table S6.

Supplementary Table S6. Primers used in this study for sexual identification and expression analysis in different developmental stages

Gene	Primer name	Sequence (5’ → 3’)	Species	Product size (bp)	
				Male	Female
Primers for sexual identification of single embryos, larvae, and pupae using genomic DNA as templates					
MoY[(1)]	MoY_F	AAATGATATAGAAGAGCATGGGATC	*Bd*, *Bc*	190	-
	MoY_R	TTCATTAATTTTTTGAATTCTGTGC			
Primers for expression analysis using cDNA as templates					
slam	Slam_F	TACACGCAAATCGAAGGTTAAGC	*Bd*, *Bc*	493	493
	Slam_R	CTCTAATTCACGTGCCAGCTC			
MoY[(1)]	MoY_F	AAATGATATAGAAGAGCATGGGATC	*Bd*, *Bc*	190	-
	MoY_R	TTCATTAATTTTTTGAATTCTGTGC			
tra[(2)]	1B-F	GAAGTTGTTATTAAGCGTAGATTCGG	*Bd*	980	626
	2B-R1	CTTTCCCGTTCGCGTTTACTATTG	*Bc*	951	599
tra-2	Tra-2_e5F	GGACCCATTGAACGAATACAAG	*Bd*, *Bc*	296	296
	Tra-2_e6bR	TTGCGACTGTGATAAGGTGAG			
dsx[(3)]	C3	GCTTAACCGCCGATCGTCAG	*Bd*, *Bc*	-	677
	Female-specific(f)	GTATTCGTTTACGACATGTTGGC			
	C4	GCTGGGGCAGATATTGAAGAG	*Bd*, *Bc*	484	-
	Male-specific(m)	CGGTACTAAGGGTTTAGTCATC			
fru	Fru_m_F	ATGTTGGCCATGTCACAAGG	*Bd*, *Bc*	506	-
	Fru_ec2_R	GCTGTTTTGAGGAACATCGG			
Primers for expression analysis using cDNA as templates					
gapdh	Gapdh_F	GTCTTCACCACAATCGATAAGGCTTCTGC	*Bd*, *Bc*	400	400
	Gapdh_R	GGAATGCCATACCAGTCAGTTTGC			

		CG			

Bd: B. dorsalis and Bc: B. correcta

(1) Primers were designed based on *BdMoY* sequence[10].

(2) Primers are from the previous study of *Bdtra* and *Bctra* sequences[11].

(3) Primers are from the previous study of *Bddsx* and *Bcdsx* sequences[12].

References

1. Liu, G., Wu, Q., Li, J., Zhang, G. & Wan, F. RNA-Mediated Knock-Down of *transformer* and *transformer 2* to Generate Male-Only Progeny in the Oriental Fruit Fly, *Bactrocera dosalis* (Hendel). *PLoS One* **10**, e0128892, doi:10.1371/journal.pone.0128892 (2015).
2. Morrow, J. L., Riegler, M., Frommer, M. & Shearman, D. C. A. Expression patterns of sex-determination genes in single male and female embryos of two *Bactrocera* fruit fly species during early development. *Insect Mol Biol* **23**, 754-767 (2014).
3. Lui, G. Q. & Wan, F. H. The gene *transformer 2* of *Bactrocera* fruit flies and its evolvement in insects. *Journal of Environmental Entomology* **4**, 742-751 (2015).
4. Sarno, F. *et al.* The gene *transformer-2* of *Anastrepha* fruit flies (Diptera, Tephritidae) and its evolution in insects. *BMC Evolutionary Biology* **10**, 140, doi:10.1186/1471-2148-10-140 (2010).
5. Schetelig, M. F., Milano, A., Saccone, G. & Handler, A. M. Male only progeny in *Anastrepha suspensa* by RNAi-induced sex reversion of chromosomal females. *Insect Biochem Mol* **42**, 51-57 (2012).
6. Salvemini, M. *et al. Ceratitis capitata transformer-2* gene is required to establish and maintain the autoregulation of *Cctra*, the master gene for female sex determination. *Int J Dev Biol* **53**, 109-120 (2009).
7. Meier, N. *et al.* Genetic control of courtship behavior in the housefly: evidence for a conserved bifurcation of the sex-determining pathway. *PLoS One* **8**, e62476-e62476, doi:10.1371/journal.pone.0062476 (2013).
8. Gailey, D. A. *et al.* Functional Conservation of the *fruitless* Male Sex-Determination Gene Across 250 Myr of Insect Evolution. *Mol Biol Evol* **23**, 633-643 (2005).
9. Bertossa, R. C., van de Zande, L. & Beukeboom, L. W. The *Fruitless* Gene in *Nasonia* Displays Complex Sex-Specific Splicing and Contains New Zinc Finger Domains. *Molecular Biology and Evolution* **26**, 1557-1569 (2009).

10. Meccariello, A. *et al. Maleness-on-the-Y* (*MoY*) orchestrates male sex determination in major agricultural fruit fly pests. *Science* **365**, 1457, doi:10.1126/science.aax1318 (2019).
11. Laohakieat, K., Aketarawong, N., Isasawin, S., Thitamadee, S. & Thanaphum, S. The study of the *transformer* gene from *Bactrocera dorsalis* and *B. correcta* with putative core promoter regions. *BMC genetics* **17**, 34-34, doi:10.1186/s12863-016-0342-0 (2016).
12. Permpoon, R., Aketarawong, N. & Thanaphum, S. Isolation and characterization of *Doublesex* homologues in the *Bactrocera* species: *B. dorsalis* (Hendel) and *B. correcta* (Bezzi) and their putative promoter regulatory regions. *Genetica* **139**, 113-127 (2011).
